# Neuroprotective and Neurite Outgrowth Stimulating Effects of New Low-Basicity 5-HT_7_ Receptor Agonists: In Vitro Study in Human Neuroblastoma SH-SY5Y Cells

**DOI:** 10.1007/s11064-024-04159-z

**Published:** 2024-06-04

**Authors:** Klaudia Jakubowska, Adam S. Hogendorf, Sławomir Gołda, Danuta Jantas

**Affiliations:** 1 Department of Experimental Neuroendocrinology, Maj Institute of Pharmacology of the Polish Academy of Sciences, Krakow, Poland; 2 Department of Medicinal Chemistry, Maj Institute of Pharmacology of the Polish Academy of Sciences, Krakow, Poland; 3 Department of Molecular Neuropharmacology, Maj Institute of Pharmacology of the Polish Academy of Sciences, Krakow, Poland

**Keywords:** Neuroprotection, 5-carboxamidotryptamine, Hydrogen peroxide, 6-hydroxydopamine, MPP +, Doxorubicin

## Abstract

There is some evidence that the serotonin receptor subtype 7 (5-HT_7_) could be new therapeutic target for neuroprotection. The aim of this study was to compare the neuroprotective and neurite outgrowth potential of new 5-HT_7_ receptor agonists (AH-494, AGH-238, AGH-194) with 5-CT (5-carboxyamidotryptamine) in human neuroblastoma SH-SY5Y cells. The results revealed that 5-HT_7_ mRNA expression was significantly higher in retinoic acid (RA)-differentiated cells when compared to undifferentiated ones and it was higher in cell cultured in neuroblastoma experimental medium (DMEM) compared to those placed in neuronal (NB) medium. Furthermore, the safety profile of compounds was favorable for all tested compounds at concentration used for neuroprotection evaluation (up to 1 μM), whereas at higher concentrations (above 10 μM) the one of the tested compounds, AGH-194 appeared to be cytotoxic. While we observed relatively modest protective effects of 5-CT and AH-494 in UN-SH-SY5Y cells cultured in DMEM, in UN-SH-SY5Y cells cultured in NB medium we found a significant reduction of H_2_O_2_-evoked cell damage by all tested 5-HT_7_ agonists. However, 5-HT_7_-mediated neuroprotection was not associated with inhibition of caspase-3 activity and was not observed in RA-SH-SY5Y cells exposed to H_2_O_2_. Furthermore, none of the tested 5-HT_7_ agonists altered the damage induced by 6-hydroxydopamine (6-OHDA), 1-methyl-4-phenylpyridinium ion (MPP +) and doxorubicin (Dox) in UN- and RA-SH-SY5Y cells cultured in NB. Finally we showed a stimulating effect of AH-494 and AGH-194 on neurite outgrowth. The obtained results provide insight into neuroprotective and neurite outgrowth potential of new 5-HT_7_ agonists.

**Supplementary Information:**

The online version contains supplementary material available at 10.1007/s11064-024-04159-z.

## Introduction

Neurodegenerative diseases (ND) are becoming an increasingly serious medical and economical issue [1]. Lack of available clinical treatments and high prevalence of ND like Alzheimer’s disease, Parkinson’s disease (PD), Huntington’s disease, multiple sclerosis and amyotrophic lateral sclerosis creates an urgent need for therapies which could attenuate or stop the disease progression [2, 3]. Although the etiology of ND differs, pathomechanisms like mitochondrial dysfunction, neuroinflammation, excitotoxicity, excessive oxidative stress and dysregulation of cell death pathways are common among them [4–7]. Despite years of research, the scientist are still trying to discover potential therapeutic targets. In this context 5-HT_7_ (serotonin type 7 receptor) seems to be an interesting target for novel therapies. 5-HT_7_ belongs to rhodopsin-like transmembrane G-protein coupled receptors (GPCRs) and was discovered as the last one from the family of serotonin receptors in 1993 [8]. It is widely expressed throughout the whole brain with the highest expression found in thalamus, hypothalamus, hippocampus and amygdala [9, 10]. The mRNA for this receptor was present also in raphe nucleus, caudate nucleus, putamen as well as in substantia nigra. Moreover, neurons, as well as astrocytes and microglia express this receptor [11]. All the splice variants of this serotonin receptor, coupled with G_s_ protein connected with stimulation of cAMP production, show high level of constitutive activity [12, 13]. By acting on G_s_ protein, adenyl cyclase (AC) is activated which results in rapid increase of intercellular cAMP level. This secondary messenger acts on protein kinase A (PKA) and causes phosphorylation of downstream effector proteins like extracellular signal-regulated kinases (ERK) and protein kinase B (Akt) [14, 15]. The signaling pathways activated by stimulation of 5-HT_7_ like protein kinase A (PKA), extracellular signal-regulated kinases (ERK) and protein kinase B (Akt) play a crucial role in maintaining proper neuron function, support the establishment of neural networks and mediate neuroprotection [12, 16]. In addition, 5-HT_7_ receptors are coupled to G_α12_ (*G protein subunit alpha 12*) activating the Rho-GEFs-Ras homolog family-Guanine Nucleotide Exchange Factor signaling and Cdc42 (*Cell division control protein 42 homolog*), which could significantly influence the cytoskeletal architecture [14, 17]. There are reports showing that Cdc42 activation by 5-HT_7_ receptor is associated with stimulation of neurites elongation in cortical, striatal and hippocampal primary cell cultures [18, 19]. Additionally, both of G-protein mediated pathways could have an impact on tyrosine receptor kinase B (TrkB) receptor expression, which has been associated with 5-HT_7_-mediated neuroprotective effects [20]. Serotonin (5-HT), through activation of 5-HT_7_, can evoke anti-inflammatory and anti-apoptotic effects. That effect has been captured as a decrease in caspase–3 and caspase–9 gene expression and was reduced after the addition of 5-HT_7_ antagonist, SB269970 [21]. Despite extensive studies, the full biological role of this receptor still remains unclear. The effects of 5-HT_7_ action has been studied using few, non-selective tool compounds, e.g.: LP-211, LP-44, LP-12, AS-19, E-55888, RA-7*,* 5-CT, 8-OH-DPAT [17, 22, 23]. 5-HT_7_ has been shown to exert a modulatory role in depression, anxiety, schizophrenia, sleep disruptions and obsessive–compulsive disorder [9, 11, 24]. It has been suggested, that 5-HT_7_ activation plays a role in neuroprotection and could increase the viability of neurons as well as have a beneficial role on neurite elongation and synaptogenesis [11, 21, 25–27]. Nevertheless, because of low selectivity, low metabolic stability and weak blood–brain barrier permeability, the search for applicable compounds is still ongoing [22, 24]. A recently emerged series of indole-imidazoles, weakly basic 5-HT_7_ agonists have been shown to exhibit high affinity and selectivity for 5-HT_7_, as well as high water solubility. Chosen compounds, such as AH-494 or AGH-192 exhibited favorable ADMET (*Absorption, Distribution, Metabolism, Excretion, Toxicity*) properties [22, 28, 29].

In this study we compared the neuroprotective and neurite outgrowth potential of known (AH-494—compound 15, AGH-238—compound 37) [29], and previously unpublished (AGH-194) 5-HT_7_ agonists with 5-carboxyamidotryptamine (5-CT) (Table 1). 5-CT is a non-selective 5-HT_7_ agonist, showing high affinity to 5-HT_1A_ [17, 30, 31]. Despite their tumor origin, human neuroblastoma SH-SY5Y cells are widely used in neurotoxicity and neuroprotection studies as a reliable and cost effective neuronal-like screening platform [32, 33]. Because these cells have dopaminergic phenotype independent of their state of differentiation, they are a common cellular model to study molecular mechanisms and new possible treatments of PD [34, 35]. It has been shown, that SH-SY5Y cells are a good model for testing biological activity of 5-HT_7_ agonists [17, 21, 27] as they express 5-HT_7_ in plasma cell membrane as well in mitochondria [36]. There is still an ongoing debate on which cell phenotype, undifferentiated or neuronally differentiated, is better for studies on neuroprotection [33, 37–39]. Thus, in our study we tested neuroprotective potential of 5-CT, AH-494, AGH-238 and AGH-194 both in undifferentiated (UN-) and retinoic acid-differentiated (RA-) SH-SY5Y cells. Hydrogen peroxide (H_2_O_2_), has been employed as an oxidative stress inducer to model cellular damage [40]. The PD type cell damage has been modeled with neurotoxins: 6- hydroxydopamine (6-OHDA) [41] and 1-methyl-4-phenylpyridine (MPP +) [42]; and a pro-apoptotic agent doxorubicin (Dox) [42]. Finally, we tested the neurite outgrowth potential of the 5-HT_7_R agonists.

**Table 1:** Chemical structure of used for the study 5-HT_7_ agonists


LAB ID	R1	R2	R3	Ki [nM]					Refs.
				5-HT_1A_	5-HT_2A_	5-HT_6_	**5-HT**_**7**_	D_2_	
AGH-238	Me	F	OMe	200	> 10,000	3802	**15**	> 10,000	Hogendorf et al., 2019
AH-494	Et	H	CONH_2_	123	> 10,000	7762	**5**	> 10,000	Hogendorf et al., 2017
AGH-194	Me	H	I	425	5427	684	**2**	> 10,000	N.P.
Reference compound									
5-CT				0.3	5012	720	**0.4**	N.D.	Leopoldo et al., 2007

*N.D.* not determined; *N.P.* not published

## Materials and Methods

### Reagents

Low basicity agonists of 5-HT_7_ were synthetized, characterized and shared for research purposes by the Department of Medicinal Chemistry, Maj Institute of Pharmacology of the Polish Academy of Sciences [22, 28, 29]. Dulbecco’s Modified Eagle’s Medium (DMEM, with high glucose and pyruvate), Neurobasal A, supplement B27 (without antioxidants), Heat Inactivated Fetal Bovine Serum (FBS), 0.25% Trypsin/EDTA solution, Penicillin–Streptomycin solution were ordered from Gibco (Invitrogen, Paisley, UK). The Cytotoxicity Detection Kit, Cell Proliferation Reagent WST-1 and Fast Start Universal Probe Master (qPCR kit) were bought from Roche Diagnostic (Mannheim, Germany). Reverse transcription kit–NG dART RT Kit were purchased from EURx Sp. z o.o. (Gdańsk, Poland). Caspase-3 substrate Ac-DEVD-AMC were purchased from Enzo Life Sciences Inc. (New York, NY, USA). All the remaining reagents were obtained from Sigma-Aldrich Chemie GmbH (Taufkirchen, Germany).

### SH-SY5Y Cell Culture

Human neuroblastoma SH-SY5Y cell line (ATCC CRL-2266, Manassas, VA, USA) was cultured in T75 flasks (SPL, Life Sciences, Korea) in DMEM medium with the addition of 10% FBS (fetal bovine serum) and 1% penicillin/streptomycin solution **(**100 U/ml penicillin and 100 μg/ml streptomycin) at the temperature of 37 °C in a saturated humidity atmosphere containing 95% air and 5% CO_2_ (ScanCell Culture Pro 170 incubator, Labogene, Denmark). After reaching 80% confluence, cells were trypsinised (0.05% trypsin/EDTA solution), counted manually (Bürker chamber) and seeded at the density of 4 × 10^4^ cells per well into 96-well plates. For differentiation, SH-SY5Y cells were seeded at a density of 2 × 10^4^ cells per well into 96-well plates in a cell culture medium supplemented with retinoic acid (RA, 10 µM). The cells have been differentiated for 6 days with the culture medium replaced every two days. One day before the experiments the culture medium was replaced by experimental medium) DMEM—DMEM containing 1% penicillin/streptomycin solution and 1% FBS or ii) NB (neuronal medium) containing Neurobasal A, supplement B27 (0.4%), streptomycin/penicillin solution (0.06 μg/ml penicillin and 0.1 μg/ml streptomycin) and L-glutamine (2 mM) in both cell-types (UN- and RA-SHSY5Y).

### Cell Treatment

For biosafety assessment of 5-HT_7_ agonists, one day before the experiments, the UN-SH-SY5Y cells were incubated in the DMEM experimental medium followed by treatment with 5-CT, AH-494, AGH-238 and AGH-194 at concentration range of 0.1–80 µM for 24 or 48 h. Biosafety tests were also conducted in UN- and RA- SH-SY5Y cells (concentration range 1–20 µM) after 24 h incubation in the NB experimental medium. Each experimental plate was used to examine cell viability (MTT reduction test) and cytotoxicity (LDH release test) simultaneously. In order to check the impact of the tested compounds on cell proliferation, SH-SY5Y cells were cultured at a density of 2 × 10^4^ cells/well in cell propagation culture medium (DMEM containing 10% FBS and 1% antibiotics). Under these conditions cells were incubated for 24–72 h with 5-CT, AH-494, AGH-238 and AGH-194 at concentration range 0.001–80 µM. Level of proliferation was measured indirectly by cell viability assay (MTT reduction test).

The neuroprotective potential of 5-CT, AH-494, AGH-238 and AGH-194 against the cell damage induced by H_2_O_2_ was determined in UN- or RA-SH-SY5Y cells placed one day before treatment in experimental medium DMEM or NB. The protective effectiveness of 5-HT_7_ agonists in other cell damage models (6-OHDA, MPP + and Dox) was tested in UN- and RA-SH-SY5Y cells cultured in NB medium. The cells were pre-treated for 30 min with 5-HT_7_ agonists (5-CT, AH-494, AGH-238 and AGH-194) at 0.01 to 1 µM. The concentration range of the 5-HT_7_ agonists was based on the affinity of each compound to 5-HT_7_ and on the previously published reports on neuroprotective properties of 5-HT_7_ agonists [20, 21, 25]. The effective concentrations and exposition time to damaging factors which evoked about 50% cell damage (measured by MTT assay) in UN- and RA-SH-SY5Y cultured in the DMEM experimental medium were optimized in our previous studies [38, 42–44]. Concentrations and incubation times used for UN- or RA-SH-SY5Y were respectively: H_2_O_2_—375 and 500 µM for 24 h.; 6-OHDA—100 and 200 µM for 24 h., MPP +− 1 and 3 mM for 48 h.; Dox—1 and 2 µM for 24 h. However, in the case of incubation of cells in NB, we observed a higher sensitivity of cells to the damaging effects of H_2_O_2_ and 6-OHDA, the concentrations were thus experimentally optimized and were as follows: H_2_O_2_—150 and 200 µM, 6-OHDA—75 and 150 µM, for UN- and RA-SH-SY5Y, respectively. For oxidative stress models an antioxidant N-acetyl-cysteine (NAC, 1 mM) was used as a positive control.

The stock solutions (10 mM) of 5-CT, AH-494, AGH-238, AGH-194 and Ac-DEVD-CHO (20 mM) were prepared in DMSO, and further diluted with distilled water. The Dox stock solution (50 mM) and the consecutive dilutions were prepared in distilled water. Prepared solutions were used over the course of this study, and stored at − 20 °C after each experiment. Stock solutions of H_2_O_2_ (100 mM), 6-OHDA (20 mM) as well as MPP + (300 mM) and their further dilutions were prepared in distilled water, freshly, just before each experiment. A stabilized 30% hydrogen peroxide solution was used to prepare the final H_2_O_2_ solution. All compounds were prepared under sterile conditions at the specified concentrations and then added to the culture medium at a volume of 1% v/v, under limited light conditions.

### Cell Viability Assays

MTT (*3-[4,5-dimethylthylthiazol-2-yl]-2,5-diphenyltetrazolium bromide*) reduction assay was used for the biosafety and cell proliferation assessments, as it was described previously [38]. The cells were incubated for 40 min with MTT reagent (at a final concentration of 0.15 mg/ml) after which the medium was removed, the product was dissolved in DMSO and the absorbance was measured at 570 nm. The SH-SY5Y cells in neuroprotection studies that were exposed to cell damaging agents became sensitive to detachment, thus, to avoid technical issues, we employed the WST-1 assay as described previously [45]. The product was soluble in cell culture medium, thus after the addition of 2.5 µl of WST-1 substrate to each well and incubation for 30 and 60 min in 37 ºC the absorbance of each sample was measured at 440 nm with reference at 630 nm. In both cell viability measurement methods, we first collected 50 µl of medium from each experimental well for the cytotoxicity assay, following the cell treatment. This was then replaced with an equal volume of fresh experimental medium before adding the substrate. The absorbance of probes was measured with multi-well plate-reader (Infinite® M200 PRO, Tecan Austria GmbH, Grodig, Austria) and data were normalized after subtraction of blank values (the absorbance of probes damaged completely by 15 min exposure to 1% TritonX_100_) and absorbance of the control cells set as 100%. The results were expressed as a mean ± S.E.M. established from 3 to 9 independent experiments with 3–5 replicates.

### Cytotoxicity Assay

Cytotoxicity was assessed by measuring the released lactate dehydrogenase (LDH) in the culture medium with Cytotoxicity Detection Kit (Roche), as described previously [38]. The absorbance of probes was measured at 490 nm with multi-well plate-reader (Infinite® M200 PRO, Tecan Austria GmbH, Grodig, Austria). The data were normalized after subtraction of blank values (the absorbance of experimental medium without cells), while the absorbance of the control cells set as 100%. The results were expressed as a mean ± S.E.M. established from 3 to 9 independent experiments with 3–5 replicates.

### Assessment of Caspase-3 Activity

For measurement of the apoptotic marker, the activity of caspase- 3, UN-SH-SY5Y cells were seeded in 6-well plates at a density of 1 × 10^6^ cells/well and incubated in the NB experimental medium. On the next day, the cells were pre-treated for 30 min with 0.01 and 0.1 µM of 5-CT, AH-494, AGH-194 or AGH-238 (0.01–1 µM) and further incubated with H_2_O_2_ (150 µM) for 9 h. As a positive control for the assay, a caspase-3 inhibitor Ac-DEVD-CHO (20 µM) was used which was added 30 min before the exposure to H_2_O_2_. The cells were lysed with ice-cold CAB (Caspase Assay Buffer) buffer and activity of caspase- 3 was measured with the fluorogenic substrate, Ac-DEVD-AMC as described in details previously [38]. The fluorescence of each experimental probe was measured using a multi-well plate-reader (Infinite® M200 PRO, Tecan Austria GmbH, Grodig, Austria) at 360 nm excitation and 460 nm emission wavelengths. The protein concentration in cell lysates was determined with the bicinchoninic acid protein assay kit (BCA1). Data were first normalized to the protein level and later calculated as a percent of control and are presented as a mean ± S.E.M. established from 2 to 6 independent experiments with 2 replicates.

### RNA Isolation, Reverse Transcription, and Quantitative PCR Analysis of 5-HT_7_ Transcripts

To determine the level of 5-HT_7_ mRNA expression, UN- and RA-SH-SY5Y cells were seeded in 24-well plates at the densities of 2 × 10^5^ and 1 × 10^5^ cells/well, respectively. Both cell phenotypes were placed in the DMEM or NB experimental medium one day before experiments. After performing live cell imaging (DIC method) cells were washed with ice-cold PBS and lysed with 500 μl of Trizol (Invitrogen, Carlsbad, CA, USA) and stored at − 20 °C. RNA isolation was performed according to manufacturer’s protocol (Invitrogen) as described previously [38]. Briefly, 100 µl of chloroform were added to each defrosted sample and then the samples were vortexed for 30 s. Then the samples were incubated on ice for 10 min and centrifuged for 20 min. at 4 °C with a relative centrifugal force of 13,000 × g. The upper phase was collected (200 µl), mixed 1:1 with isopropanol, vortexed and incubated for another 30 min at − 20 °C. Then, the samples were centrifuged for 30 min at 4 °C with a relative centrifugal force of 13,000 × g, the supernatants were removed and the remaining RNA pellets were washed with 1 ml 70% ice-cold ethanol (− 20 °C). After the removal of the supernatant, the pellet was dried and suspended in 20 µl of RNAze free water and denatured for 5 min at 65 °C. The concentration of RNA was measured using NanoDrop ND-1000 Spectrometer (NanoDrop Technologies Inc., Montchanin, DE, USA). Reverse transcription was performed with 2 µg RNA and NG dART RT Kit (EURx) according to manufacturer recommendation at 37 °C for 60 min. qPCR reaction was performed by use of TaqMan Assay-On-Demand probes (*HTR7-* Hs04194798_s1, *HPRT-1* Hs02800695) and Fast Start Universal Probe Master (Roche) kit, according to the recommendations of the manufacturer, using CFX96 device (BioRad). cDNAs were diluted 1:10 with pharmaceutical purified water and approximately 90 ng of the cDNA synthesized from the RNA template from an individual sample was used for each reaction. The expression of the hypoxanthine guanine phosphoribosyltransferase 1 (*HPRT1*) transcript, which remained stable both in UN- and RA-SH-SY5Y was used as the reference transcript. Cycle threshold values (Ct) were calculated automatically by a program and the amounts of mRNA for each transcript were calculated as 2^−dCt^. To compare the results between the cell phenotypes and types of experimental medium, all the obtained results were presented as fold changes in the mean expression of all the experimental groups in a given assay, relatively to the measured reference transcript (2^−dCt^). The data are presented as a mean ± S.E.M. established from 3 independent experiments with 3 replicates.

### Light Microscopy Imaging and Neurite Outgrowth Analysis

Light microscopy differential interference contrast (DIC) method was used to show the neuroprotective effects of the tested agonists *vs.* reference compound (NAC) in the model of damage caused by H_2_O_2_. Next, the impact of cell differentiation and type of experimental medium on cell morphology was demonstrated, as well the impact of the tested 5-HT_7_ agonists on neurite length. For evaluation of 5-HT_7_ agonists action on neurite outgrowth, the SH-SY5Y cells were seeded in 24-well plates at the density of 1 × 10^5^ cells/well. Twenty four hours after cell seeding, 5-CT, AH-494, AGH-238 and AGH-194 were added to the cells at 0.01–1 µM concentrations. RA (10 µM) was used as a positive control for neurite outgrowth assay. Imaging was performed 24 and 48 h after cell treatment with an inverted microscope AxioObserver.Z1 (Carl Zeiss, Germany) and images were taken by a black and white camera AxioCam MRm (Carl Zeiss, Germany). For each experimental group, 4 microphotographs were taken per well. For neurite length estimation, a randomly chosen set of 10 neurites from each image were measured by ImageJ program with *Simple Neurite Tracer* plug-in. Results of the measurements made after 24 and 48 h were first calculated as a percent of growth from time 0 h for each experiment. In the next stage, the lengths were calculated as the differences to the controls for particular time points and they were subjected to a statistical analysis. The data were presented as means ± S.E.M. established from 2 independent experiments with 2 replicates.

### Statistical Analysis

Data were analyzed using the Statistica 13 software (StatSoft Inc., Tulsa, OK, USA). To demonstrate statistical significance, the analysis of variance (one- or two-way ANOVA) and posthoc Duncan's test for multiple comparisons were used with assumed p < 0.05.

## Results

### The Impact of Differentiation and the Type of Experimental Medium on the Morphology and Viability of UN- and RA-SH-SY5Y Cells

**Fig. 1:**
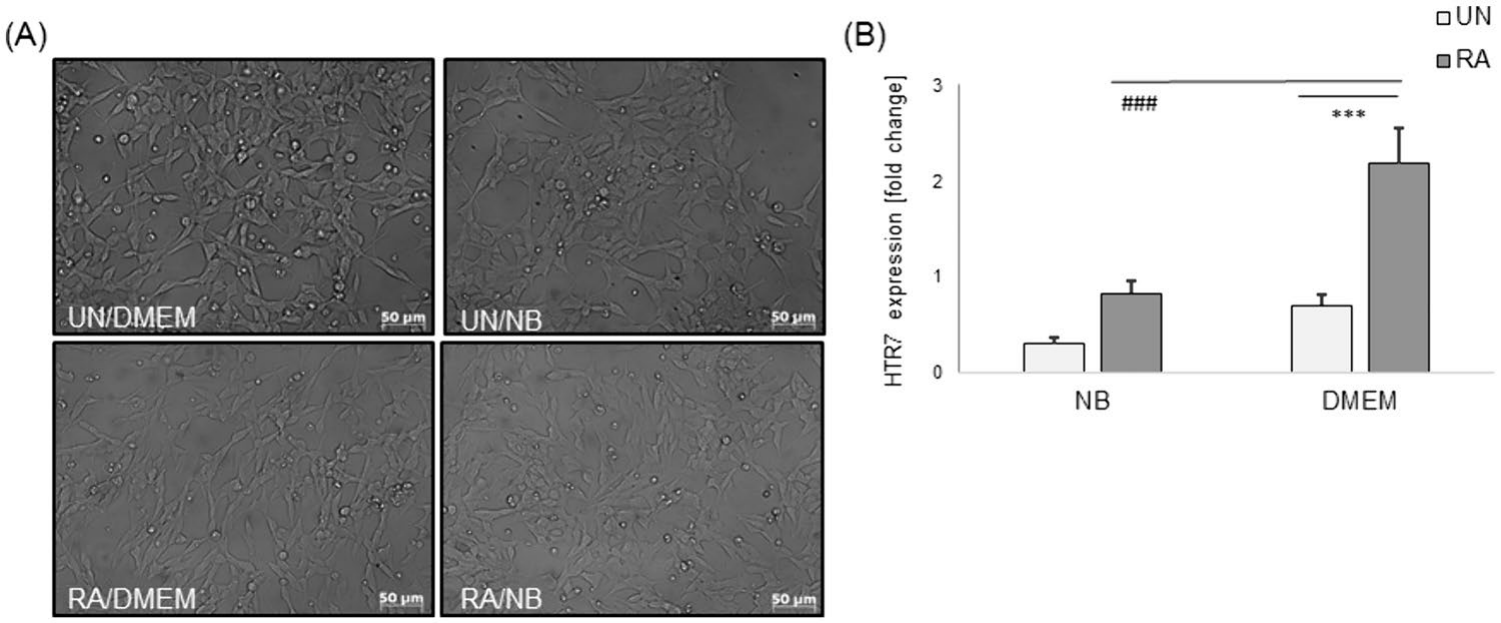
**A** DIC (Differential Contrast Images) microphotographs of undifferentiated (UN) and retinoic acid-differentiated (RA) SH-SY5Y cells after 24 h incubation in DMEM or NB experimental medium. **B** Expression of mRNA for 5-HT_7_ in undifferentiated (UN-) and retinoic acid (RA-)-differentiated SH-SY5Y cells incubated in neuroblastoma (DMEM) and neuronal (NB) experimental medium. The cells were cultured for 24 h in DMEM or NB experimental medium. mRNA level for 5-HT_7_ receptor as well as housekeeping gene (*HPRT1*) were measured by RT-qPCR method. Results are presented as a fold change from the average 5-HT_7_ expression from all groups. Data from three independent experiments in triplicates were analyzed by two-way ANOVA and Duncan’s posthoc test. ^***^p < 0.001 UN *vs.* RA cell’s phenotypes; ^###^p < 0.001 DMEM vs NB experimental medium

SH-SY5Y cells had a decreased proliferation rate during differentiation process induced by RA. They became more elongated and polarized in both types of the experimental medium (DMEM or NB), compared to the corresponding groups of UN-SH-SY5Y cells (Fig. 1A). After 24 h incubation of cells in the NB medium we noticed an increased plate adherence of the cells compared to incubation in DMEM, regardless of the cell phenotype. Additionally, an increase in the number of apoptotic cells was observed in UN-SH-SY5Y cells cultured in DMEM when compared to RA-SH-SY5Y cells maintained in the same type of experimental medium as well as when compared to UN-SH-SY5Y cells cultured in the NB medium. However, in SH-SY5Y cells incubated in NB medium no changes in the number of apoptotic cells were observed between UN- and RA-SH-SY5Y cells (Fig. 1A).

### The Impact of Differentiation and the Type of Experimental Medium on 5-HT_7_ Receptor mRNA Expression in SH-SY5Y Cells

The RA-SH-SY5Y cells incubated in DMEM medium showed a significantly higher level of mRNA expression for the 5-HT_7_ receptor than the cells maintained in NB. However, UN-SH-SY5Y showed no significant difference in mRNA expression depending on the type of experimental medium used. Furthermore, in case of cells cultured in DMEM there was significantly higher level of *HTR7* expression in RA-SH-SY5Y compared to UN-SH-SY5Y (Fig. 1B). Additionally for UN- and RA-SH-SY5Y incubated in the NB medium the additional statistical analysis was carried out using the t-student test, which showed a significant increase in *HTR7* expression in RA-SH-SY5Y cells, when compared UN-SH-SY5Y cells (p < 0.01).

### Evaluation of 5-HT_7_ Agonists Biosafety Profile in UN-SH-SY5Y Cells Cultured in DMEM and NB Experimental Medium

**Fig. 2:**
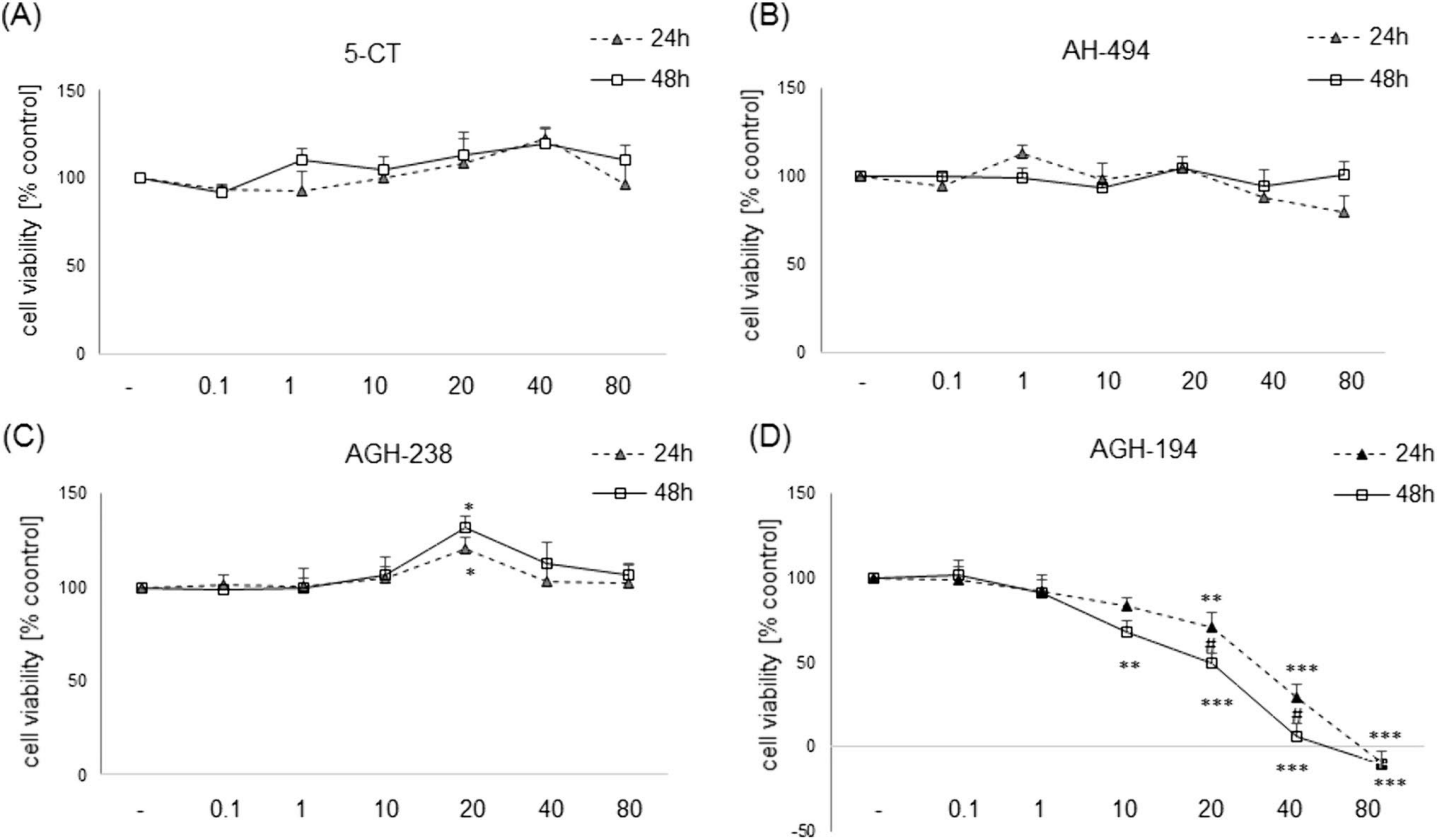
Biosafety profile of 5-HT_7_ agonists in UN-SH-SY5Y in the DMEM experimental medium. Cells were incubated for 24 and 48 h with 5-CT, AH-494, AGH-238 and AGH-194 at concentration range of 0.1–80 µM. Viability of cells was measured with MTT reduction. Results were normalized to control group and are presented as a mean ± S.E.M. Data from 3–6 independent experiments were analyzed two-way ANOVA and Duncan’s posthoc test. ^*^p < 0.05, ^**^p < 0.01 and ^***^p < 0.001 *vs.* control groups of particular time points; ^#^p < 0.05 and ^###^p < 0.001 24 h *vs.* 48 h for particular concentrations

**Fig. 3:**
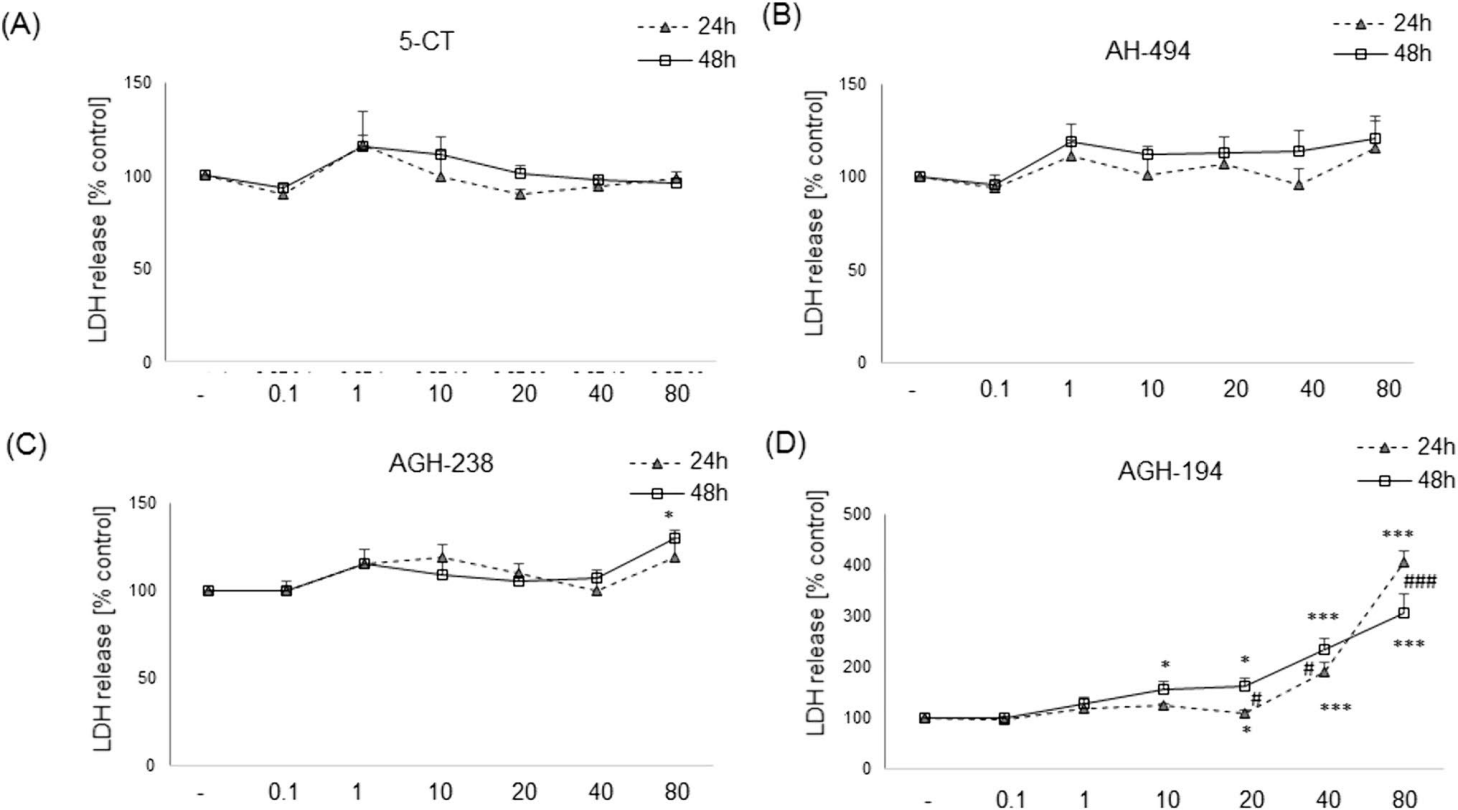
Biosafety profile of 5-HT_7_ agonists in UN-SH-SY5Y in the DMEM experimental medium. Cells were incubated for 24 and 48 h with 5-CT, AH-494, AGH-238 and AGH-194 at concentration range of 0.1–80 µM. Cytotoxicity was measured by LDH release assay. Results were normalized to control group and are presented as a mean ± S.E.M. Data from 3–6 independent experiments were analyzed two-way ANOVA and Duncan’s posthoc test. ^*^p < 0.05, ^**^p < 0.01 and ^***^p < 0.001 *vs.* control groups of particular time points; ^#^p < 0.05 and ^###^p < 0.001 24 h *vs.* 48 h for particular concentrations

The incubation of UN-SH-SY5Y cells maintained in DMEM medium for 24 as 48 h with 5-CT and AH-494 in wide range of concentrations (0.1–80 µM) did not affect cell viability and cytotoxicity when compared to control groups (Figs. 2A, B, 3A, B). AGH-238 at concentration 20 µM showed an increase in cell viability after 24 h (~ 20%) and 48 h (~ 30%) (Fig. 2C), and in LDH release test this compound at concentration 80 μM after 48 h evoked a significant (~ 30%) increase in cytotoxicity level (Fig. 3C). For AGH-194 we observed a concentration- and time-dependent decrease in cell viability (~ 30–100%) with maximum damage of cells evoked by its higher concentrations (40 and 80 μM) (Fig. 2D). Moreover, for concentrations 20 and 40 µM of AGH-194 there was a greater decrease in cell viability after 48 h compared to 24 h. In the cytotoxicity assay an increase in LDH release was observed for concentrations 40 and 80 µM (~ 2–4- times) after 24 h incubation but also for concentrations 10–80 µM (~ 1.5–3 times) after 48 h. In addition for 20 and 40 µM of AGH-194 there was a significant increase in LDH release after 48 h compared to the level obtained after 24 h. Whereas in the case of 80 µM higher level of released LDH was observed after 24 h than after 48 h (Fig. 3D).

Twenty four hours incubation of UN-SH-SY5Y cells maintained in NB medium with 5-CT, AGH-238 and AH-494 at concentration range 1–20 µM did not affect cell viability (Table 2). However, AGH-194 at concentration of 20 μM evoked a significant decrease of cell viability at (~ 20%) (Table 2). An additional statistical analysis by t-test for AGH-194 at concentration 20 μM between cells maintained in DMEM and NB experimental medium revealed no significant differences between tested groups (p = 0.477).

**Table 2:** Biosafety profile of 5-HT_7_ agonists in UN-SH-SY5Y cells in NB experimental medium

	UN-SH-SY5Y	RA-SH-SY5Y
control	100.00	100.00
5-CT 1 5-CT 10 5-CT 20	106.79 ± 5.54	99.67 ± 6.67
	114.1 ± 13.79	98.63 ± 3.94
	105.53 ± 15.85	92.65 ± 5.64
AH-494 1 AH-494 10 AH-494 20	116.22 ± 18.20	99.19 ± 3.80
	108.76 ± 7.00	99.02 ± 10.37
	112.46 ± 7.33	109.47 ± 3.01
AGH-238 1 AGH-238 10 AGH-238 20	86.53 ± 9.57	98.07 ± 5.09
	85.74 ± 11.80	100.22 ± 3.33
	88.38 ± 8.69	100.52 ± 6.81
AGH-194 1 AGH-194 10 AGH-194 20	102.57 ± 0.54	106.96 ± 2.98
	97.27 ± 2.68	84.26 ± 4.07 *
	81.99 ± 7.16 *	77.05 ± 2.86 **

The cells were incubated for 24 h with 5-CT, AH-494, AGH-238 and AGH-194 in at concentration range 1–20 µM. Cell viability was measured by MTT reduction test. Results were normalized to control group and was are presented as mean ± S.E.M. Data from two independent experiments were analyzed by one-way ANOVA and Duncan’s post hoc test. *p < 0.05 and **p < 0.01 *vs.* control group

### The Effect of 5-HT_7_ Agonists on SH-SY5Y Cell Proliferation

**Fig. 4:**
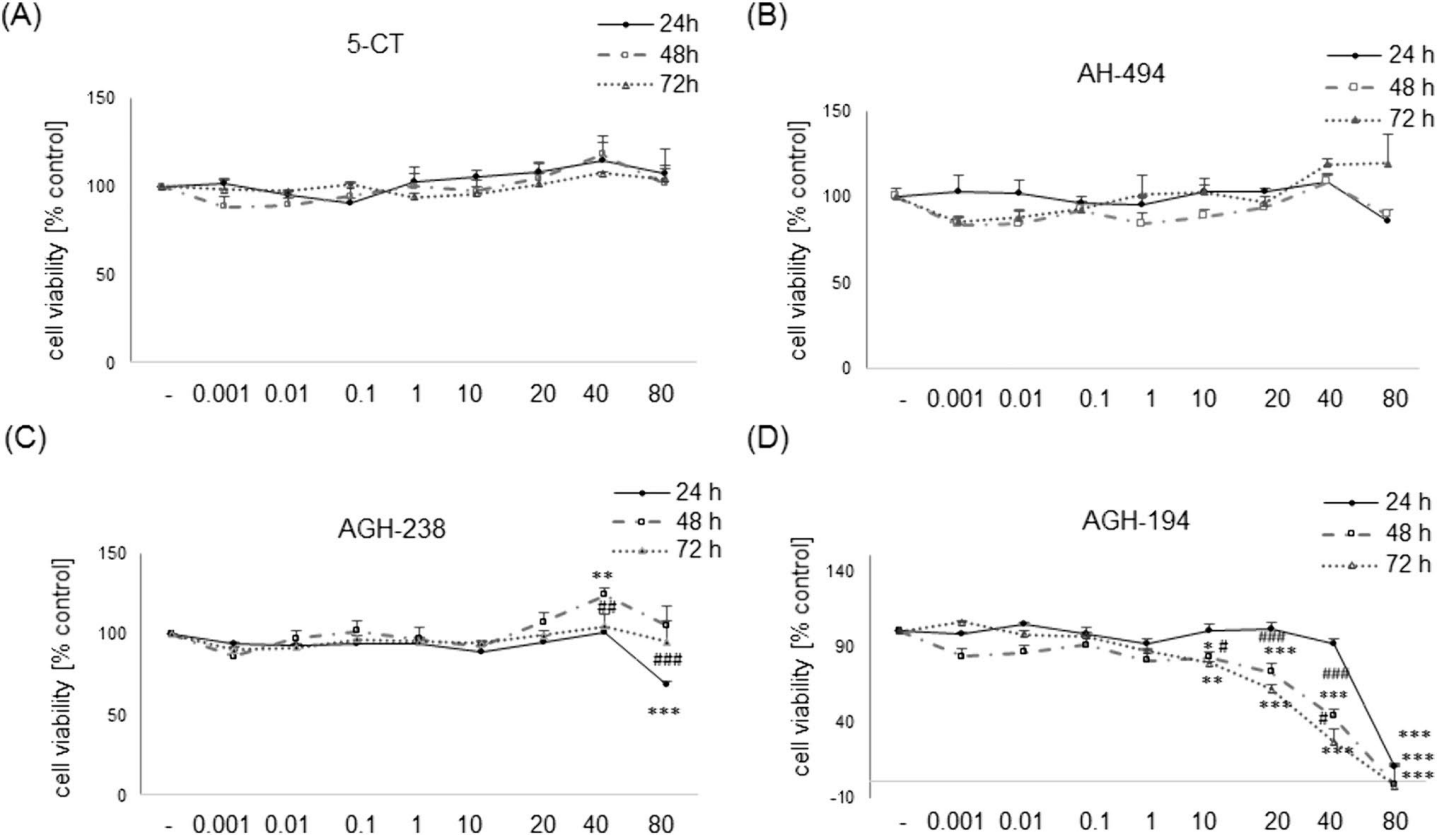
The effect of 5-HT_7_ agonists on SH-SY5Y cell proliferation. The cells were incubated for 24, 48 and 72 h with 5-CT, AH-494, AGH-238 and AGH-194 at concentration range of 0.001–80 µM in DMEM culture medium containing 10% FBS. Cell proliferation was measured indirectly by MTT reduction test. Results were normalized as a percentage of control and are presented as a mean ± S.E.M. Data from 3–6 independent experiments were analyzed by two-way ANOVA and Duncan’s posthoc test. ^*^p < 0.05, ^**^p < 0.01 and ^***^p < 0.001 *vs.* control groups of particular time points; ^#^p < 0.05, ^##^p < 0.01 and ^###^p < 0.001 *vs.* indicated time points for particular concentrations

We did not observe any changes in cell proliferation rate measured by MTT reduction test after 24, 48 and 72 h of incubation of SH-SY5Y cells with 5-CT and AH-494 in wide range of concentrations (0.001–80 µM) in DMEM cell culture medium containing 10% FBS (Figs. 4A and B). However incubation with AGH-238 at concentration of 40 µM showed a transient increase in cell viability (~ 25%) after 48 h (Fig. 4C). This effect was significantly different when compared to 24 or 72 h time points. The same compound at concentration of 80 µM after 24 h induced a transient viability decrease (~ 30%), and that effect disappeared after longer incubation times (48 and 72 h) (Fig. 4C). AGH-194 evoked a time- and a concentration-dependent reduction in cell viability (Fig. 4D). Maximal cell damage was observed for concentration 80 µM after 24 h and this effect was similar after 48 h and 72 h. Concentrations 10–40 µM of AGH-194 evoked a concentration-dependent decrease in cell viability (~ 20–60%) after 48 h and these effects were significantly higher to relevant concentrations at 24 h time point. We noticed that AGH-194 at concentration 40 µM induced significantly higher reduction in cell viability (~ 20%) after 72 h when compared to 48 h (Fig. 4D).

### Evaluation of Neuroprotective Potential of 5-HT_7_ Agonists Against Cell Damage Induced by H_2_O_2_ in SH-SY5Y Cells Cultured in DMEM and NB Experimental Medium

While UN-SH-SY5Y cells were incubated with H_2_O_2_ (375 μM) in DMEM experimental medium, the damage factor induced about 50% cell viability decrease as well over 3.5-fold increase in LDH release when compared to control group. These effects were significantly decreased by antioxidant NAC (Table S1). In UN-SH-SY5Y cells cultured in NB experimental medium we found that H_2_O_2_ (150 μM) evoked around 30% decrease in cell viability and about fourfold increase in LDH level which was significantly decreased by NAC (Table S1). Similar observations were done for RA-SH-SY5Y cells cultured in NB experimental medium where H_2_O_2_ (200 µM) evoked about 35% decrease in cell viability and fourfold increase in LDH level which in the former assay were significantly reversed by NAC (Table S2). The biochemical results for UN- and RA-SH-SY5Y cells incubated with DMEM or NB medium and treated for 24 h with H_2_O_2_ and NAC was also confirmed at morphological level using DIC light microscopy method. After treatment of cells with H_2_O_2_ we observed an increased number of rounded cells without protrusions with weaker adhesion to the surface which were prevented by NAC (Fig. S1).

**Fig. 5:**
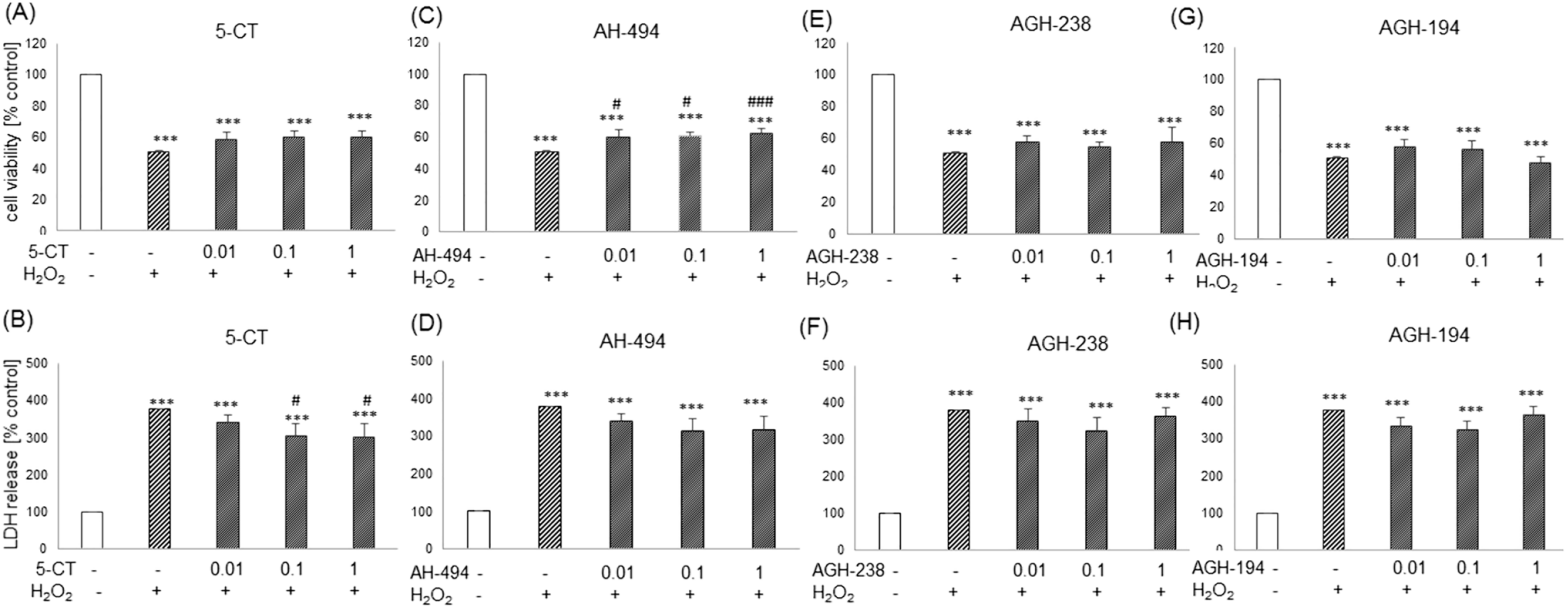
Neuroprotective effects of 5-HT_7_ agonists against the H_2_O_2_-inducedcell damage in UN-SH-SY5Y cells cultured in DMEM experimental medium. The cells were pre-treated for 30 min with 5-CT, AH-494, AGH-238 and AGH-194 at concentration range of 0.01–1 µM followed by 24 h treatment with H_2_O_2_ (375 µM). Cell viability and cytotoxicity were measured by WST-1 and LDH release assays, respectively. Results were normalized to control group and are presented as a mean ± S.E.M. Data from 4–5 independent experiments were analyzed by one-way ANOVA and Duncan’s posthoc test. ^***^p < 0.001 *vs.* control group; ^#^p < 0.05 and ^###^p < 0.001 *vs.* H_2_O_2_ group

In UN-SH-SY5Y cells incubated in DMEM medium 5-CT at concentrations of 0.01–1 µM did not affect the H_2_O_2_-induced decrease in cell viability (Fig. 5A), however in cytotoxicity test, concentrations of 0.1 and 1 µM of this compound significantly decreased the level of LDH induced by H_2_O_2_ (Fig. 5B). AH-494 at concentrations of 0.01–1 µM showed small but statistically significant increase (~ 10%) in cell viability when compared to the H_2_O_2_-treated cells (Fig. 5C), whereas in cytotoxicity assay we did not observe any protective effects for AH-494 (Fig. 5D). The incubation of cells with AGH-238 as well as AGH-194 at concentrations of 0.01–1 µM did not significantly affect the extent of cell damage evoked by H_2_O_2_ in both used screening assays (WST-1 and LDH release tests). (Figs. 5E–H).

**Fig. 6:**
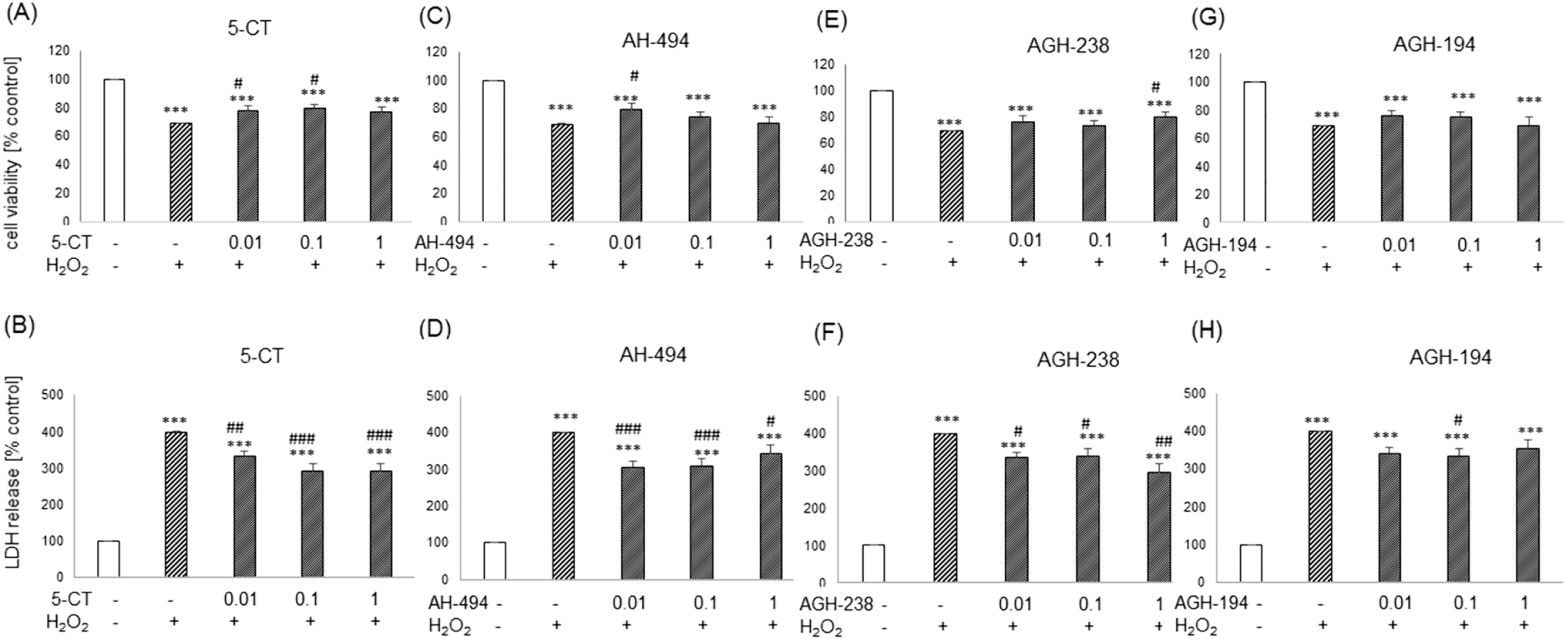
Neuroprotective effects of 5-HT_7_ agonists against the H_2_O_2_-inducedcell damage in UN-SH-SY5Y cells cultured in NB experimental medium. The cells were pre-treated for 30 min with 5-CT, AH-494, AGH-238 and AGH-194 at concentration range of 0.01–1 µM followed by 24 h treatment with H_2_O_2_ (150 µM). Cell viability and cell cytotoxicity were measured with WST-1 and LDH release assays, respectively. Results were normalized to control group and are presented as a mean ± S.E.M. Data from 6 to 9 independent experiments were analyzed by one-way ANOVA and Duncan’s posthoc test. ^***^p < 0.001 *vs.* control group; ^##^p < 0.01 and ^###^p < 0.001 vs. H_2_O_2_ group

**Fig. 7:**
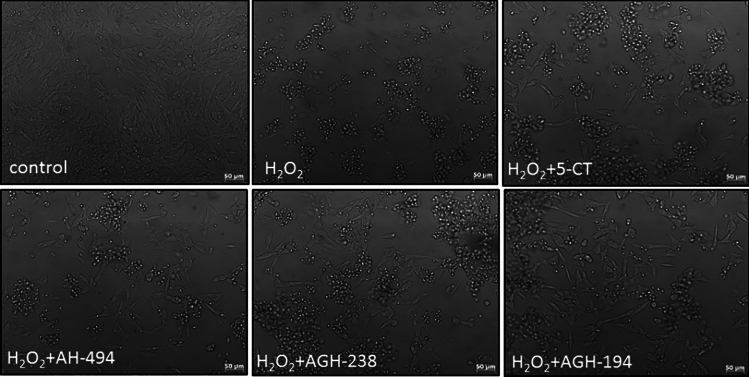
DIC (Differential Contrast Images) microphotographs of UN-SH-SY5Y cells cultured in NB experimental medium and treated with 5-HT_7_ agonists and H_2_O_2_. Cells were pre-treated for 30 min with 5-CT, AH-494, AGH-238 and AGH-194 at concentration of 0.1 µM followed by 24 h of treatment with H_2_O_2_ (150 µM)

5-CT at concentrations of 0.01 and 0.1 µM significantly increased (9–11%) UN-SH-SY5Y cell viability cultured in NB medium when compared to H_2_O_2_ group (Fig. 6A). The same compound at concentrations of 0.01–1 µM decreased level of H_2_O_2_-stimulated LDH release about 68–110% (Fig. 6B). AH-494 at concentration of 0.01 µM significantly increased (~ 10%) cell viability as well as decreased amount of released LDH (94–57%) in comparison to results from H_2_O_2_ group (Figs. 6C–D). For compound AGH-238 at concentration of 1 µM an significant increase in cell viability was observed (Fig. 6E) as also decrease in cytotoxicity for concentrations of 0.01–1 µM (64–105%) when compared to the H_2_O_2_ treated group (Fig. 6F). AGH-194 at the level of cell viability assessment did not affect the extend of cell damage evoked by H_2_O_2_ (Fig. 6G)_,_ although in cytotoxicity assay, at concentration of 0.1 µM it partially attenuated the H_2_O_2_-evoked increase in LDH release (Fig. 6H). Neuroprotective effects of 5-HT_7_ agonists found in biochemical assays were confirmed also at morphological level (Fig. 7) where we observed partial improvement of cell morphology after treatment with 0.1 µM 5-CT, AH-494, AGH-238 or AGH-194 in comparison to the H_2_O_2_ group.

In RA-SH-SY5Y cells cultured in NB medium we did not observe any protective effects of 5-CT, AH-494, AGH-238 and AGH-194 at concentrations of 0.01–1 µM against the cell damage induced by H_2_O_2_ (200 µM) as confirmed by cell viability and cytotoxicity assays (Table 3). Furthermore, in the LDH release test there was a significant increase of cytotoxicity evoked by AGH-238 at concentration of 1 µM when compared to H_2_O_2_ group (Table 3).

**Table 3:** Neuroprotective effects of 5-HT_7_ agonists against the H_2_O_2_-induced cell damage in RA-SH-SY5Y cells cultured in NB experimental medium

	WST-1	LDH
control	100.00	100.00
H_2_O_2_	65.93 ± 0.36 ***	406.67 ± 0.67 ***
5-CT 0.01 + 5-CT 0.1 + 5-CT 1 +	71.10 ± 2.32 *** 71.52 ± 2.42 *** 65.27 ± 3.41 ***	386.74 ± 17.81 *** 387.25 ± 19.37 *** 373.50 ± 14.19 ***
AH-494 0.01 + AH-494 0.1 + AH-494 1 +	65.06 ± 3.80 *** 64.95 ± 3.17 *** 66.22 ± 3.85 ***	399.96 ± 17.07 *** 393.17 ± 16.90 *** 394.25 ± 25.75 ***
AGH-238 0.01 + AGH-238 0.1 + AGH-238 1 +	65.67 ± 7.61 *** 66.44 ± 5.02 *** 57.23 ± 8.34 ***	420.69 ± 20.84 *** 399.50 ± 32.24 *** 479.45 ± 34.62 ***, #
AGH-194 0.01 + AGH-194 0.1 + AGH-194 1 +	62.35 ± 6.88 *** 56.30 ± 7.41 *** 54.13 ± 9.72 ***	406.72 ± 14.59 *** 436.58 ± 48.09 *** 469.69 ± 49.05 ***
N	6	4–6

Cells were pre-treated for 30 min with 5-CT, AH-494, AGH-238 and AGH-194 at concentration range 0.01–1 µM followed by 24 h of treatment with H_2_O_2_ (200 µM). Cell viability and cell cytotoxicity were measured with WST-1 and LDH release assays, respectively. Results were normalized to control groups and are presented as the mean ± S.E.M. Data from 4–6 independent experiments were analyzed by one-way ANOVA and Duncan’s posthoc test. ^***^p < 0.001 *vs.* control group; ^#^p < 0.05 *vs.* H_2_O_2_ group

### Effects of 5-HT7 Agonists on H_2_O_2_-Induced Caspase–3 Activity in UN-SH-SY5Y Cells Cultured in NB Experimental Medium

**Fig. 8:**
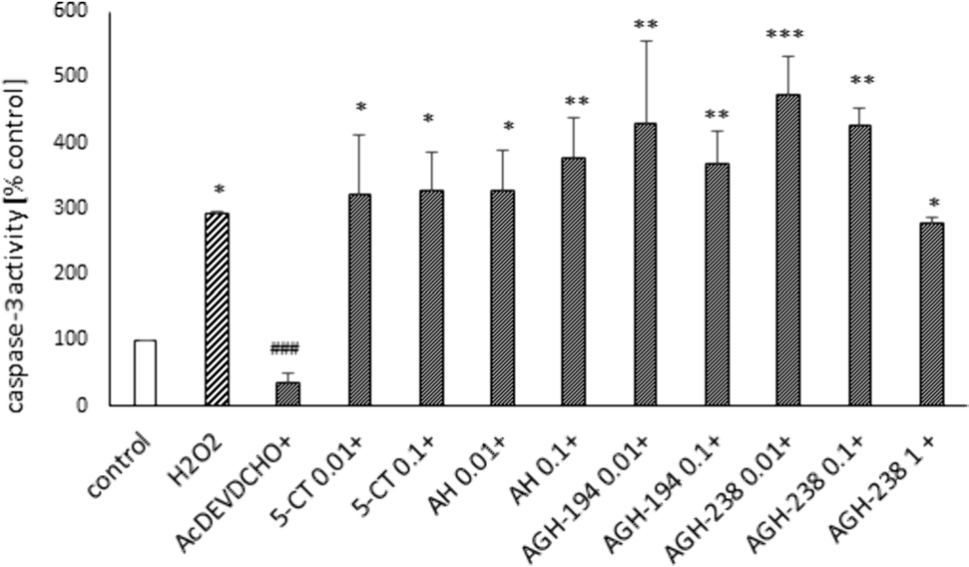
The impact of 5-HT_7_ agonists on caspase–3 activity induced by H_2_O_2_ in UN-SH-SY5Y cells cultured in NB culture medium. The cells were pre-treated for 30 min with 5-CT, AH-494 and AGH-194 at concentrations of 0.01 and 0.1 µM and with AGH-238 at concentrations of 0.1 and 1 µM followed by 9 h of treatment with H_2_O_2_ (150 µM). Caspase-3 inhibitor, Ac-DEVD-CHO (20 µM) was used as positive control for the assay. Results were normalized to control and are presented as a mean ± S.E.M. Data from 2–6 independent experiments were analyzed by one-way ANOVA and Duncan’s posthoc test. ^*^p < 0.05, ^**^p < 0.01 and ^***^p < 0.001 *vs.* control group; ^###^p < 0.001 *vs*. H_2_O_2_ group

In order to explore some of mechanisms which could be engaged in 5-HT_7_-mediated neuroprotection against the H_2_O_2_-evoked cell damage, we measured activity of the main apoptotic executor protease, caspase-3 [38]. Incubation of UN-SH-SY5Y cells cultured in NB experimental medium for 9 h with H_2_O_2_ (150 µM) induced approximately fourfold increase in caspase–3 activity which was fully inhibited by caspase–3 inhibitor, Ac-DEVD-CHO (20 µM) but not by any of the tested 5-HT_7_ agonists (Fig. 8).

### Evaluation of 5-HT_7_ Agonists Neuroprotective Potential in Other Cells Damage Models in UN- and RA-SH-SY5Y Cultured in NB Experimental Medium

In order to verify a neuroprotective action of 5-HT_7_ agonist in the PD cellular model we incubated UN-SH-SY5Y cells with 6-OHDA (75 µM) in NB medium which induced approximately 55% decrease of viability in comparison to control group, and this effect was significantly attenuated by NAC (Table S2). In case of RA-SH-SY5Y cells after 24 h of treatment with 6-OHDA (150 µM) 35% decrease of cell viability was observed and this effect was also significantly reversed by NAC (Table S2).

**Fig. 9:**
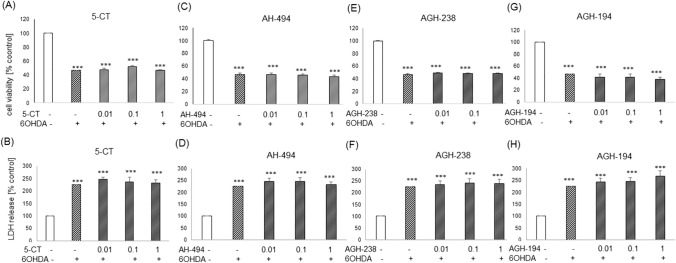
The effects of 5-HT_7_ agonists against the 6-OHDA-evoked cell damage in UN-SH-SY5Y cells cultured in NB experimental medium. Cells were incubated for 30 min with 5-CT, AH-494, AGH-238 and AGH-194 at concentration range of 0.01–1 µM followed by 24 h exposure to 6-OHDA (75 µM). Cell viability and cell cytotoxicity were measured with WST-1 and LDH release assays, respectively. Results of absorbance measurement for each group were normalized to control and presented as a mean ± S.E.M of 6–8 independent experiments. Data were analyzed by one-way ANOVA and Duncan’s post hoc test. ^***^p < 0.001 *vs*. control;

Twenty four hours of treatment of UN-SH-SY5Y cells cultured in NB medium with 5-CT, AH-494, AGH-238 and AGH-194 at concentrations of 0.01–1 µM and neurotoxin 6-OHDA did not change the cell viability (Figs. 9A, C, E, G) and cytotoxicity (Figs. 9B, D, F, H). Similar results we observed in RA-SH-SY5Y cells in the same experimental medium (NB) where 5-HT_7_ agonists did not show any protection against the 6-OHDA-induced cell damage (Table S3).

Forty eight hours of treatment of UN-SH-SY5Y cells with MPP + (1 mM) evoked about 50% reduction in cell viability which was not changed by 5-HT_7_ agonists at any tested concentration (0.01–1 µM) (Table S4). Similar results we found in RA-SH-SY5Y cells where 5-HT_7_ agonist did not show any protection against the MPP +−induced cell damage (Table S4).

Twenty four hours treatment of UN- and RA-SH-SY5Y cells in NB experimental medium with Dox (1 and 2 µM for UN- and RA-SH-SY5Y, respectively) evoked approximately 40–50% decrease in cell viability as measured by WST-1 test (Table S5). 5-CT, AH-494, AGH-238 and AGH-194 in none of the concentrations used (0.01–1 µM) had a significant effect on UN- and RA-SH-SY5Y cells viability decrease evoked by Dox (Table S5).

### The Effects of 5-HT_7_ Agonists on Neurite Outgrowth in SH-SY5Y Cells

**Fig. 10:**
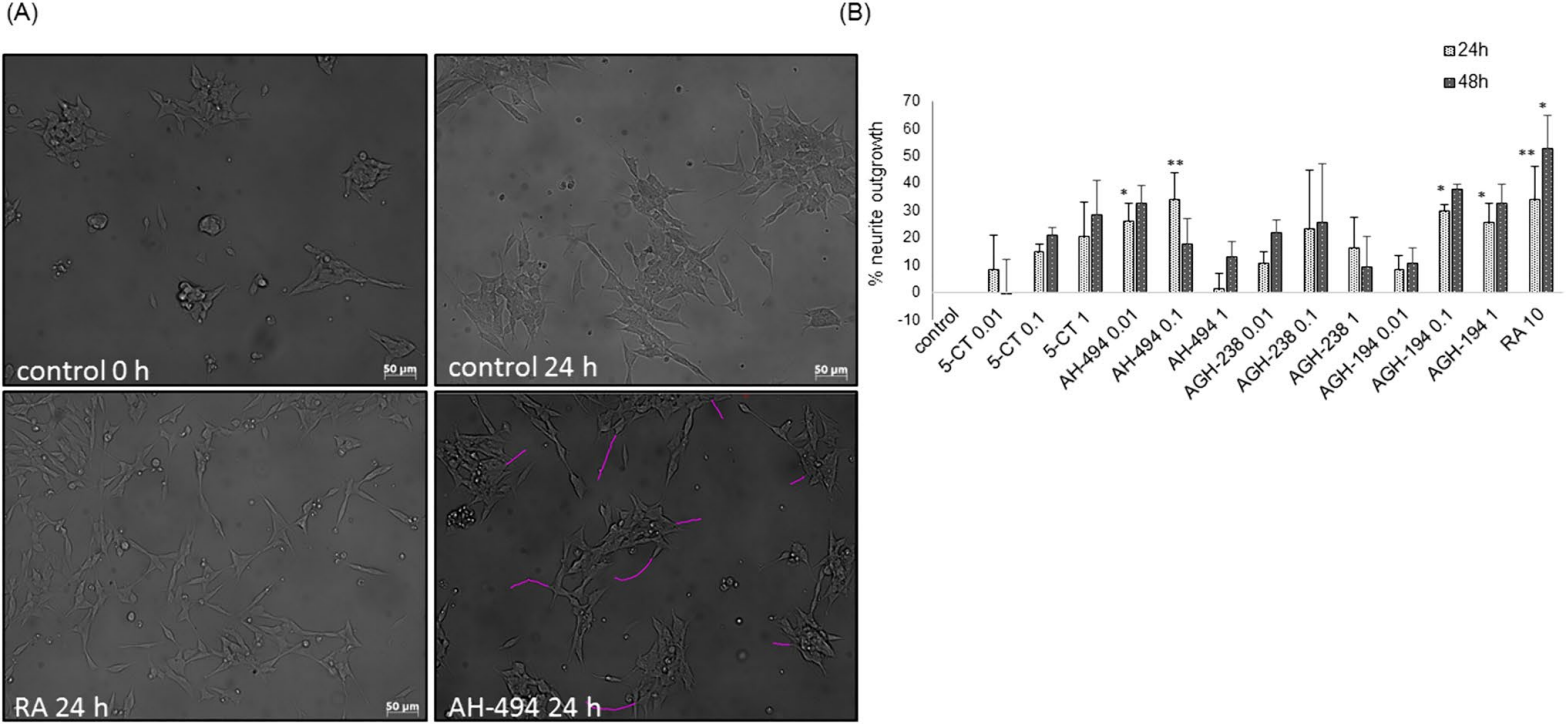
**A** DIC (Differential Contrast Images) microphotographs of SH-SY5Y cells after 24 h of treatment with AH-494 (0.1 µM). Retinoic acid (RA, 10 µM) was used as positive control for the assay. Neurite outgrowth was measured in ImageJ program in the way which is presented for AH-494 compound (24 h). **B** The effects of 5-HT_7_ agonists on neurite outgrowth in SH-SY5Y cells. Cells were incubated for 24 or 48 h with 5-CT, AH-494, AGH-238 and AGH-194 at concentration range of 0.01–1 µM. Retinoic acid (RA, 10 µM) was used as a positive control. Neurite outgrowth was measured in ImageJ program with *Simple Neurite Tracer* plug-in. Results are expressed as percentage of neurite outgrowth relative to control for each time point and are presented as a mean ± S.E.M. Data for each time point were analyzed separately by one-way ANOVA and Duncan’s post hoc test. ^*^p < 0.05 and ^**^p < 0.01 *vs.* control group for each time point

To examine the impact of tested 5-HT_7_ agonists on neurite outgrowth, the SH-SY5Y cells were treated for 24 and 48 h with 5-CT, AH-494, AGH-238 and AGH-194 at concentrations of 0.01–1 µM. 5-CT and AGH-238 at concentrations of 0.01–1 µM did not significantly affect neurite outgrowth in SH-SY5Y cells when compared to control group at any of tested time points. AH-494 and AGH-194 at concentrations of 0.01 and 0.1 µM after 24 h of incubation significantly increased the neurite length to the level which was achieved by positive control RA (10 µM). After 48 h of treatment we still observed an increased neurite length by RA, but we did not find significant effects for any of tested 5-HT_7_ agonists. However, some tendency in increase of this parameter could be noted for AH-494 (0.01 µM) and AGH-194 (0.1 and 1 µM) (Fig. 10).

## Discussion

According to the Ehrlich’s concept of a ‘magic bullet’, there’s a demand to find selective chemical compounds, which could induce specific actions within cell signaling via specific receptors. These compounds may be used as research tools or therapeutics. In contrast, commonly used protein ligands often act non-specifically and could cause undesirable, negative effects on the organism as a whole [46–49]. It is often relatively easy to find selective antagonist of a protein of interest, compared to a selective agonist. 5-HT_7_ receptor emerged as a putative molecular target for neurological disorders such as anxiety, depression, neurodegenerative disorders and pain [9, 23, 24]. Although pyrroloimidazoles have been reported in the context of neurite outgrowth stimulation, they have not been linked to, and it is highly doubtful, that their effects could be mediated by 5-HT_7_ signalling [50]. It should be underlined that currently there have been only a few experimental studies which investigated the neuroprotective potential of the 5-HT_7_ receptor [21, 27, 51, 52]. For that reason obtaining any new data regarding 5-HT_7_ contribution in attenuating nervous system disorders could be profitable. So far for in vitro and in vivo research dedicated to 5-HT_7_ mechanism of action, SB269970—specific antagonist/inverse agonist of 5-HT_7_ [53] and commercially available agonist 5-CT were tested for examining the pharmacological activation of the receptor [54–56]. In case of 5-CT there are some issues that should be taken into consideration. 5-CT was shown to be non-selective, despite the high affinity for 5-HT_7_ [14, 17] and the results thus obtained by use of this substance could be misinterpreted in relation to its mechanism of action. Throughout the years, many research groups synthesized and examined compounds acting on 5-HT_7_ as they were trying to find agonists which have better pharmacokinetic parameters and also high selectivity to 5-HT_7_ [17, 57–59]. So far no compounds acting through 5-HT_7_ were found and considered to be used in clinic [24, 31, 60, 61]. Thus we tested in the context of neuroprotective and neurite outgrowth properties the three new, low basicity 5-HT_7_ agonists: AH-494, AGH-238 and AGH-194, that have a high affinity to 5-HT_7_ and good selectivity over 5-HT_1A_. First, we measured the expression of 5-HT_7_ in our model, the human neuroblastoma SH-SY5Y cells. We showed the impact of cell differentiation state and the type of the experimental medium on the expression of transcript for 5-HT_7_. We confirmed previous results showing higher expression of *HTR7* in differentiated SH-SY5Y when compared to undifferentiated ones [25] and showed for the first time the impact of the type of used experimental medium on this target. We observed a higher level of 5-HT_7_ transcript in cells incubated in neuroblastoma dedicated medium (DMEM with low serum content) in comparison to cells placed in neuronal medium (NB). It cannot be excluded that stimulation of the expression might be a cell endogenous response for trophic factors deprivation, associated with the serum reduction from 10 to 1%, whereas in NB medium trophic factors are provided by B27 supplement [62].

The second step was undertaken to establish the biosafety profile of the tested 5-HT_7_ agonists. In this context, the highest level of cytotoxicity as well as the effect on cell viability decrease was found for AGH-194. It was shown that this compound evoked cell death in UN-SH-SY5Y cells incubated both in DMEM and NB at concentrations above 10 µM. A decrease in cell viability was also observed for AGH-194 at concentrations of 10 µM and 20 µM in RA-SH-SY5Y, which confirmed the high cytotoxicity of this compound. It should be noted that after differentiation, SH-SY5Y cells usually show higher resistance to various cell damaging factors [38, 43] which was not the case with AGH-194. Biosafety profile of the other tested 5-HT_7_ agonists (AH-494, AGH-238 and 5-CT) up to 80 µM was found to be favorable for UN- and RA-SH-SY5Y cells cultured in DMEM experimental medium. Previous data for AH-494 (0.1–100 µM) in HEK-293 and SH-SY5Y showed no cytotoxic effect of this compound [22], which is in line with our findings. However, in HepG2 cells concentration of 100 µM of AH-494 evoked significant reduction in cell viability, pointing that possible detrimental effects could be the cell type-dependent [22]. It is worth to mention that the experiments done by Latacz et al. [22] were performed under typical cell culturing conditions for proliferating cells (DMEM + 10% FBS), which could make cells more resistant to potentially harmful compounds. In contrast, our results were obtained in conditions of reduced serum content (DMEM + 1% FBS) which could make cells more vulnerable to potential toxicity of the tested agents. In our study, AGH-238 induced transient increase of cell viability at concentration of 20 µM in UN-SH-SY5Y cells cultured in DMEM experimental medium, which probably is connected with the impact of this compound on cell proliferation, since our cellular model is of tumor origin. For that reason we also checked the impact of 5-HT_7_ agonists on SH-SY5Y cells proliferation under standard cell culturing conditions (DMEM + 10% FBS). We observed that 5-CT as well as AH-494 up to 80 µM did not affect the cell proliferation (measured indirectly by MTT test). For AGH-238 at concentration of 40 µM a transient increase in cell viability/proliferation was observed at 48 h of incubation which is partially consistent with the effect observed in results of biosafety profile for this compound, whereas at concentration of 80 µM after 24 h this compound evoked transient viability decrease, which disappeared during longer incubation time (48 h and 72 h). This effect could explained as restarting the proliferation process by cells which survived the initial exposure to the compound and/or by compound deactivation after longer time of incubation. To confirm the aforementioned hypothesis, further experiments would be necessary, where the proliferation rate would be measured with more specific markers like BrdU or Ki67 [63]. The highest decrease of cell viability that was time and concentration dependent, and interpreted as a decrease of proliferation was obtained for AGH-194, where at 80 µM, almost total reduction of cell viability for each of the tested time points was observed. For the lower concentrations (10–40 µM) there was a significant decrease of cell viability (~ 20–60%) after 48 h and 72 h in comparison to the 24 h treatment. The observed changes at the higher concentrations were probably caused by cellular death rather than proliferation suppression. When testing biosafety prolife of 5-HT_7_ agonists in UN- and RA-SH-SY5Y cell cultured in NB medium at concentration range up to 20 µM for 24 h we noticed a significant reduction of cell viability by AGH-194 (at concentration 20 and 10–20 µM for UN- and RA-SH-SY5Y cells, respectively) but not by the other tested compounds. The above findings prove, that AGH-194 shows greater cytotoxicity than other the tested 5-HT_7_ ligands under various cell culture conditions at least in human SH-SY5Y cells, which could prohibit its potential future use in the clinic. Those effects could potentially be attributed to the increased lipophilicity of AGH-194 versus AGH-238 or AH-494.

Since we observed the impact of the cell differentiation state as well the type of experimental medium on 5-HT_7_ expression, thus we examined the potential neuroprotective effects of 5-HT_7_ compounds in UN- and RA-SH-SY5Y cells cultured in DMEM and NB experimental medium. For this purpose we used an inducer of oxidative stress, H_2_O_2_, as well as neurotoxins that mimic PD, like 6-OHDA and MPP + as well as a pro-apoptotic factor, Dox. By their use we could model cellular damage which mimics different pathological mechanisms of PD like apoptosis, necrosis, autophagy, oxytosis, pyroptosis, necroptosis [7, 64, 65]. H_2_O_2_ is broadly used in cellular models as an oxidative stress inducer for testing a protective potential of medicinal substances [66]. In that type of cell damage we were able to demonstrate for the first time neuroprotection mediated by 5-HT_7_ agonists and that effect was dependent on the cell differentiation state as well on the type of experimental medium. We observed some protection for 5-CT (0.1–1 µM) in LDH release test and for AH-494 (0.01–1 µM) in cell viability test against the H_2_O_2_-evoked detrimental changes in UN-SH-SY5Y cells cultured in DMEM experimental medium. Since these results were not confirmed in both biochemical tests thus this neuroprotective effect could be interpreted as biologically negligible. However, in the NB medium we found a significant protection by 5-CT, AH-494 and AGH-238 in both cell viability and cytotoxicity assays. For AGH-194 we observed only a decrease of the H_2_O_2_-evoked LDH release but no effects on the cell viability which suggests lower neuroprotective potential of this compound. It is worth to highlight that all of the observed changes after 5-HT_7_ treatment were partial and none of tested compound completely reduced detrimental effects induced by H_2_O_2_ to the level mediated by antioxidant, NAC. Referring to these neuroprotective effects mediated by 5-HT_7_ agonists, it can be concluded that LDH test is more sensitive for detection of neuroprotective response of 5-HT_7_ ligands which were also confirmed by our cell imaging studies. Interestingly, in RA-SH-SY5Y we did not find any protection by 5-HT_7_ agonists under both types of experimental medium which suggest that mechanisms of protection mediated by this compounds could be mimicked by RA during the process of cell differentiation where activation of prosurvival pathways like PI3K/AKT and ERK 1/2 takes place [37]. Similar observation was done by us for ligands of metabotropic glutamate receptors group II or III which were protective against staurosporine, Dox or MPP + -induced cell damage in UN- but not in RA-SH-SY5Y [38, 43]. It should be noted that the protective effects observed after 5-HT_7_ agonists treatment against the H_2_O_2_-evoked cell damage were not positively correlated with the level of expression of 5-HT_7_ mRNA in particular cell types. Tested compounds had stronger protective properties in UN-SH-SY5Y cells placed in NB medium than DMEM, while no protection was observed in RA-SH-SY5Y under any experimental condition, when *HTR7* expression was the highest in RA-SH-SY5Y cells cultured in DMEM medium. It can refer to the fact that the obtained mRNA level for the transcript could not be translated to significant protein levels in cells [66], but this assumption needs to be experimentally verified. An additive factor that can contribute to divergent effects could be non-specific action of ligands, especially 5-CT which can activate either 5-HT_7_ and also 5-HT_1A_ receptor [58]. What is more, these receptors can dimerize with each other creating complexes, what can additively cause problems with interpretation of effects [14]. Nevertheless, there were some research outcomes which indicated protective properties of commercially available 5-HT_7_ ligands [9, 17, 20, 24, 67], thus our results are supporting already existing literature data. Regarding previous reports on a neuroprotective potential of 5-HT_7_ agonists, Vasefi et al. [27] showed that 24 h incubation of primary hippocampal cells with LP12 (300 nM) prevented neurotoxicity caused by NMDA activation and this effect was dependent on PDGFβ (*Platelet-derived growth factor β*) receptor kinase activation. Yuksel et al. [21] examined the protective action of LP-44 (10^−10^, 10^−9^, 10^−8^ mM) in an oxidative stress model induced by a high concentration of glutamate (80 mM) in SH-SY5Y cells. This group also observed an increased expression of 5-HT_7_ receptor after glutamate exposure. These results can suggest that the receptor expression could increase in unfavorable cell conditions, which is consistent with our observations, where under serum deprivation (medium DMEM 1%) mRNA level for 5-HT_7_ was significantly increased compared to the NB medium. Moreover, on the basis of the above findings it can be noticed that the neuroprotective effect was induced by low concentrations of 5-HT_7_ agonists which is in line with our data. In the future studies, the specificity of 5-HT_7_ activation of observed protective effects should be taken into consideration as well as intracellular pathways which could be responsible for these effects. Regarding the latter, in our study we showed that tested 5-HT_7_ agonists did not decrease the level of caspase–3 activity induced by H_2_O_2_. This is in contrast to the findings of Yuksel et al. [21], who showed that LP-44-mediated neuroprotection against glutamate-evoked oxytosis in SH-SY5Y cells was associated with caspase-3 and caspase-9 inhibition. It should be underlined that protective effects without simultaneous decrease of caspase-3 activity were observed in our previous study when we tested the effectiveness of agonists of metabotropic glutamatergic receptors group II and III against cell damage evoked by proapoptotic factors (staurosporine and Dox) [43]. Since in these models we observed the participation of caspase- 3-independent mechanism coupled with inhibition of AIF (*apoptosis inducing factor*) translocation to nucleus [43], it is not excluded that a similar mechanism is involved in the case of 5-HT_7_ agonist action against the H_2_O_2_ evoked cell damage. It was shown in SH-SY5Y and neuronal cells that after H_2_O_2_ exposure there is an induction of AIF translocation [43, 68]. Moreover, the inhibition of calcium dependent proteases, calpains or lysosomal enzyme, cathepsin D [69] or the inhibition of necroprotsis [44] could be the other possible mechanisms responsible for 5-HT_7_ mediated protection against H_2_O_2_.

The neuroprotective action of 5-HT_7_ agonists observed in this study seems to be limited to the model of oxidative stress induced by H_2_O_2_, since we did not find any attenuation by 5-CT, AH-494, AGH-238 or AGH-194 of the cell damage evoked by 6-OHDA, MPP + and Dox both in UN- and RA-SH-SY5Y cells, cultured in NB experimental medium. It suggests that the neuroprotective effects of 5-HT_7_ agonists are strongly dependent on the type of cell damage and they can involve the protection mechanisms that are specific to the cascade of events leading to cellular death induced by H_2_O_2._ It should be underlined that the previous findings also pointed that effects mediated by 5-HT_7_ receptor in the CNS may be evoked by either agonists and antagonists (or inverse agonists) in dependence on the type of cell damage or the used experimental model [70]. For example SB-269970 (5-HT_7_ selective antagonist) increased the production of dopaminergic cells in rat’s midbrain neutrospheres whereas activation of 5-HT_7_ receptor by 8-OH-DPAT inhibited this process [71]. While neuronal damage was modeled by 6-OHDA in rat’s prefrontal cortex, 5-HT_7_ agonist AS-19 showed antidepressant effects which were reversed by SB-269970 [72]. What is interesting, while 6-OHDA evoked lesions in lateral habenula, this effect was reversed by SB-269970 which showed also antidepressant properties, whereas AS-19 induced prodepressive behavior in rats [73]. Moreover it is believed that antagonists of 5-HT_7_ are promising for the treatment of schizophrenia or epilepsy [9]. SB-269970 is commonly used to block the protective effects of 5-HT_7_ agonists [21] and in the future it is advisable to check if this compound inhibits the effects of the herein tested agonists. To this end we did not find any protection mediated by SB-269970 (0.1–10 µM) against cell damage induced by H_2_O_2_ and 6-OHDA in UN- and RA-SH-SY5Y cells cultured in NB experimental medium (data not shown).

At the final stage of the study, we investigated if the tested 5-HT_7_ ligands have an impact on neurite outgrowth in SH-SY5Y cells. Two of the tested compounds (AGH-194 and AH-494) demonstrated beneficial properties in that aspect and their action was stronger compared to the commercially available agonist 5-CT. These effects were observed only after 24 h incubation with the agonists, which suggests that their action was transient. As a positive control we used RA (10 μM), which significantly increased the neurite length in each investigated time point (24 h and 48 h). The effect of 5-HT_7_ stimulation on neurite elongation was previously examined and confirmed by Chang et al. [25], who incubated SH-SY5Y cells with 5-HT (1 μM) and LP211 (0.1 and 1 μM), and the effect of these compounds was inhibited by SB-269970. But what differs our work from their is the analysis, because they calculated only the percentage of neurons with a length of neurites greater than 50 μm. In comparison to that we analyzed elongation for specific time points. Moreover, it is possible that this effect we observed only in the case of two compounds, is 5-HT_7_ dependent, because 5-CT which has a similar affinity to 5-HT_7_ and 5-HT_1A_ did not demonstrate any action promoting neurite outgrowth in our experimental setting. Since the previous studies demonstrated that changes in the cytoskeleton are dependent on G_α12_ and Cdc42 activation after 5-CT (100 nM) stimulation in primary hippocampal cell cultures [74], their possible involvement in the observed beneficial effects of 5-HT_7_ agonists in SH-SY5Y cells should be further investigated.

## Conclusions

Our data showed that SH-SY5Y cells are a suitable cellular model for testing the biological activity of 5-HT_7_ agonists, because they express 5-HT_7_ receptors, which is cell phenotype and experimental medium composition dependent. Furthermore, we showed that the new low-basicity 5-HT_7_ agonists (AH-494, AGH-238 and AGH-194) as well as the commercially available 5-CT show some neuroprotective effects against H_2_O_2_-evoked cell damage in SH-SY5Y cells and this effect is also dependent on the cell phenotype and experimental medium composition. The observed neuroprotective effects of 5-HT_7_ agonists were not associated with the inhibition of caspase–3 activity. Among the tested compounds, AH-494 appeared to have the greatest protective potential, whereas AGH-194 the lowest one. None of the examined compounds showed neuroprotective properties in cellular damage models evoked by 6-OHDA, MPP + and Dox in SH-SY5Y cells. Finally, the two of the tested 5-HT_7_ agonists (AH-494 and AGH-194) promoted neurite outgrowth. The results obtained in SH-SY5Y cells provide new insights in the context of neuroprotective and neurite outgrowth potential of the new 5-HT_7_ agonists, which should be verified in the future in animal studies.

### Supplementary Information

Below is the link to the electronic supplementary material.

Supplementary file1 (DOCX 1128 KB)

## Data Availability

The raw data that support the findings of this study are available upon reasonable request from the corresponding author.

## Abbreviations

6-OHDA: 6-Hydroxydopamine
DIC: Differential interference contrast
Dox: Doxorubicin
H_2_O_2_: Hydrogen peroxide
LDH: Lactate dehydrogenase
MPP +: 1-Methyl-4-phenylpyridine
MTT: 3-[4,5-Dimethylthiazol-2-yl]-2,5-diphenyltetrazolium bromide
ND: Neurodegenerative diseases
PD: Parkinson’s disease
RA: Retinoic acid
RA-SH-SY5Y: Retinoic acid-differentiated SH-SY5Y cells
ROS: Reactive oxygen species
UN-SH-SY5Y: Undifferentiated SH-SY5Y cells

## References

[1] Temple S (2023). Advancing cell therapy for neurodegenerative diseases. Cell Stem Cell.

[2] Durães F, Pinto M, Sousa E (2018). Old drugs as new treatments for neurodegenerative diseases. Pharmaceuticals.

[3] Hansson O (2021). Biomarkers for neurodegenerative diseases. Nat Med.

[4] Crabbé M, Dirkx N, Casteels C (2019). Excitotoxic neurodegeneration is associated with a focal decrease in metabotropic glutamate receptor type 5 availability: an *in vivo* PET imaging study. Sci Rep.

[5] Dugger BN, Dickson DW (2017). Pathology of neurodegenerative diseases. Cold Spring Harb Perspect Biol.

[6] Emerit J, Edeas M, Bricaire F (2004). Neurodegenerative diseases and oxidative stress. Biomed Pharmacother.

[7] Ghavami S, Shojaei S, Yeganeh B (2014). Autophagy and apoptosis dysfunction in neurodegenerative disorders. Prog Neurobiol.

[8] Bard JA, Zgombick J, Adham N (1993). Cloning of a novel human serotonin receptor (5-HT_7_) positively linked to adenylate cyclase. J Biol Chem.

[9] Blattner KM, Canney DJ, Pippin DA, Blass BE (2018). Pharmacology and therapeutic potential of the 5-HT_7_ receptor. ACS Chem Neurosci.

[10] Ciranna L, Catania MV (2014). 5-HT_7_ receptors as modulators of neuronal excitability, synaptic transmission and plasticity: physiological role and possible implications in autism spectrum disorders. Front Cell Neurosci.

[11] Quintero-Villegas A, Valdés-Ferrer SI (2022). Central nervous system effects of 5-HT_7_ receptors: a potential target for neurodegenerative diseases. Mol Med.

[12] Huang S, Xu P, Shen DD (2022). GPCRs steer Gi and Gs selectivity via TM5-TM6 switches as revealed by structures of serotonin receptors. Mol Cell.

[13] Krobert KA, Levy FO (2002). The human 5-HT_7_ serotonin receptor splice variants: constitutive activity and inverse agonist effects. Br J Pharmacol.

[14] Guseva D, Wirth A, Ponimaskin E (2014). Cellular mechanisms of the 5-HT_7_ receptor-mediated signaling. Front Behav Neurosci.

[15] Norum JH, Hart K, Levy FO (2003). Ras-dependent ERK activation by the human Gs-coupled serotonin receptors 5-HT_4(b)_ and 5-HT_7(a)_. J Biol Chem.

[16] Gellynck E, Heyninck K, Andressen KW, Haegeman G, Levy FO, Vanhoenacker P, Van Craenenbroeck K (2013). The serotonin 5-HT 7 receptors: two decades of research. Exp Brain Res.

[17] Di Pilato P, Niso M, Adriani W (2014). Selective agonists for serotonin 7 (5-HT_7_) receptor and their applications in preclinical models: an overview. Rev Neurosci.

[18] Speranza L, Giuliano T, Volpicelli F (2015). Activation of 5-HT_7_ receptor stimulates neurite elongation through mTOR, Cdc42 and actin filaments dynamics. Front Behav Neurosci.

[19] Volpicelli F, Speranza L, di Porzio U (2014). The serotonin receptor 7 and the structural plasticity of brain circuits. Front Behav Neurosci.

[20] Samarajeewa A, Goldemann L, Vasefi MS (2014). 5-HT_7_ receptor activation promotes an increase in TrkB receptor expression and phosphorylation. Front Behav Neurosci.

[21] Yuksel TN, Yayla M, Halici Z (2019). Protective effect of 5-HT_7_ receptor activation against glutamate-induced neurotoxicity in human neuroblastoma SH-SY5Y cells via antioxidative and antiapoptotic pathways. Neurotoxicol Teratol.

[22] Latacz G, Hogendorf AS, Hogendorf A (2018). Search for a 5-CT alternative. In vitro and in vivo evaluation of novel pharmacological tools: 3-(1-alkyl-1 H-imidazol-5-yl)-1 H-indole-5-carboxamides, low-basicity 5-HT 7 receptor agonists. Med Chem Comm.

[23] Nikiforuk A (2015). Targeting the serotonin 5-HT_7_ receptor in the search for treatments for CNS disorders: rationale and progress to date. CNS Drugs.

[24] Fukuyama K, Motomura E, Okada M (2023). Therapeutic Potential and Limitation of Serotonin Type 7 Receptor Modulation. Int J Mol Sci.

[25] Chang WY, Yang YT, She MP (2022). 5-HT_7_ receptor-dependent intestinal neurite outgrowth contributes to visceral hypersensitivity in irritable bowel syndrome. Lab Invest.

[26] Rojas PS, Neira D, Muñoz M (2014). Serotonin (5-HT) regulates neurite outgrowth through 5-HT_1A_ and 5-HT_7_ receptors in cultured hippocampal neurons. J Neurosci Res.

[27] Vasefi MS, Kruk JS, Heikkila JJ (2013). 5-Hydroxytryptamine type 7 receptor neuroprotection against NMDA-induced excitotoxicity is PDGFβ receptor dependent. J Neurochem.

[28] Hogendorf AS, Hogendorf A, Kurczab R (2017). Low-basicity 5-HT_7_ receptor agonists synthesized using the van Leusen multicomponent protocol. Sci Rep.

[29] Hogendorf AS, Hogendorf A, Popiołek-Barczyk K (2019). Fluorinated indole-imidazole conjugates: Selective orally bioavailable 5-HT_7_ receptor low-basicity agonists, potential neuropathic painkillers. Eur J Med Chem.

[30] Cowen DS, Johnson-Farley NN, Travkina T (2005). 5-HT_1A_ receptors couple to activation of Akt, but not extracellular-regulated kinase (ERK), in cultured hippocampal neurons. J Neurochem.

[31] Leopoldo M, Lacivita E, Berardi F (2011). Serotonin 5-HT_7_ receptor agents: structure-activity relationships and potential therapeutic applications in central nervous system disorders. Pharmacol Ther.

[32] Forster JI, Köglsberger S, Trefois C (2016). Characterization of differentiated SH-SY5Y as neuronal screening model reveals increased oxidative vulnerability. J Biomol Screen.

[33] Ioghen OC, Ceafalan LC, Popescu BO (2023). SH-SY5Y cell line in vitro models for Parkinson disease research—old practice for new trends. J Integr Neurosci.

[34] Krishna A, Biryukov M, Trefois C (2014). Systems genomics evaluation of the SH-SY5Y neuroblastoma cell line as a model for Parkinson’s disease. BMC Genomics.

[35] Xicoy H, Wieringa B, Martens GJ (2017). The SH-SY5Y cell line in Parkinson’s disease research: a systematic review. Mol Neurodegener.

[36] Tempio A, Niso M, Laera L (2020). Mitochondrial membranes of human sh-sy5y neuroblastoma cells express serotonin 5-HT_7_ receptor. Int J Mol Sci.

[37] Cheung YT, Lau WKW, Yu MS (2009). Effects of all-trans-retinoic acid on human SH-SY5Y neuroblastoma as in vitro model in neurotoxicity research. Neurotoxicology.

[38] Jantas D, Greda A, Golda S (2014). Neuroprotective effects of metabotropic glutamate receptor group II and III activators against MPP (+)-induced cell death in human neuroblastoma SH-SY5Y cells: the impact of cell differentiation state. Neuropharmacology.

[39] Pandey M, Karmakar V, Majie A (2024). The SH-SY5Y cell line: a valuable tool for Parkinson’s disease drug discovery. Expert Opin Drug Discov.

[40] Ruffels J, Griffin M, Dickenson JM (2004). Activation of ERK1/2, JNK and PKB by hydrogen peroxide in human SH-SY5Y neuroblastoma cells: role of ERK1/2 in H_2_O_2_-induced cell death. Eur J Pharmacol.

[41] Gomez-Lazaro M, Bonekamp NA, Galindo MF (2008). 6-Hydroxydopamine (6-OHDA) induces Drp1-dependent mitochondrial fragmentation in SH-SY5Y cells. Free Radical Biol Med.

[42] Jantas D, Pytel M, Mozrzymas JW (2008). The attenuating effect of memantine on staurosporine-, salsolinol-and doxorubicin-induced apoptosis in human neuroblastoma SH-SY5Y cells. Neurochem Int.

[43] Jantas D, Greda A, Leskiewicz M (2015). Neuroprotective effects of mGluR II and III activators against staurosporine-and doxorubicin-induced cellular injury in SH-SY5Y cells: new evidence for a mechanism involving inhibition of AIF translocation. Neurochem Int.

[44] Jantas D, Chwastek J, Grygier B (2020). Neuroprotective effects of necrostatin-1 against oxidative stress–induced cell damage: an involvement of cathepsin d inhibition. Neurotox Res.

[45] Jantas D, Malarz J, Le TN (2021). Neuroprotective properties of kempferol derivatives from maesa membranacea against oxidative stress-induced cell damage: An association with cathepsin d inhibition and pi3k/akt activation. Int J Mol Sci.

[46] Buckley JS, Salpeter SR (2015). A risk-benefit assessment of dementia medications: systematic review of the evidence. Drugs Aging.

[47] Carta M, Carlsson T, Kirik D, Björklund A (2007). Dopamine released from 5-HT terminals is the cause of L-DOPA-induced dyskinesia in parkinsonian rats. Brain.

[48] Nagatsu T, Sawada M (2009). L-dopa therapy for Parkinson's disease: past, present, and future. Parkinsonism Relat Disord.

[49] Wang SM, Han C, Bahk WM (2018). Addressing the side effects of contemporary antidepressant drugs: a comprehensive review. Chonnam Med J.

[50] Beck B, Leppert CA, Mueller BK, Dçmling A (2006). Discovery of pyrroloimidazoles as agents stimulating neurite outgrowth. QSAR Comb Sci.

[51] Lee J, Avramets D, Jeon B (2021). Modulation of serotonin receptors in neurodevelopmental disorders: Focus on 5-HT_7_ receptor. Molecules.

[52] Modica MN, Lacivita E, Intagliata S (2018). Structure–activity relationships and therapeutic potentials of 5-HT_7_ receptor ligands: an update. J Med Chem.

[53] Hagan JJ, Price GW, Jeffrey P (2000). Characterization of SB-269970-A, a selective 5-HT7 receptor antagonist. Br J Pharmacol.

[54] Guscott MR, Egan E, Cook GP (2003). The hypothermic effect of 5-CT in mice is mediated through the 5-HT_7_ receptor. Neuropharmacology.

[55] Hedlund PB, Sutcliffe JG (2004). Functional, molecular and pharmacological advances in 5-HT_7_ receptor research. Trends Pharmacol Sci.

[56] Laplante P, Diorio J, Meaney MJ (2002). Serotonin regulates hippocampal glucocorticoid receptor expression via a 5-HT_7_ receptor. Dev Brain Res.

[57] Kułaga D, Drabczyk AK, Satała G (2022). Design and synthesis of new potent 5-HT_7_ receptor ligands as a candidate for the treatment of central nervous system diseases. Eur J Med Chem.

[58] Medina RA, Sallander J, Benhamu B (2009). Synthesis of new serotonin 5-HT7 receptor ligands. Determinants of 5-HT7/5-HT1A receptor selectivity. J Med Chem.

[59] Na YH, Hong SH, Lee JH (2008). Novel quinazolinone derivatives as 5-HT_7_ receptor ligands. Bioorg Med Chem.

[60] Kucwaj-Brysz K, Baltrukevich H, Czarnota K (2021). Chemical update on the potential for serotonin 5-HT_6_ and 5-HT_7_ receptor agents in the treatment of Alzheimer’s disease. Bioorg Med Chem Lett.

[61] Matthys A, Haegeman G, Van Craenenbroeck K (2011). Role of the 5-HT_7_ receptor in the central nervous system: from current status to future perspectives. Mol Neurobiol.

[62] Martin ER, Gandawijaya J, Oguro-Ando A (2022). A novel method for generating glutamatergic SH-SY5Y neuron-like cells utilizing B-27 supplement. Front Pharmacol.

[63] Kee N, Sivalingam S, Boonstra R, Wojtowicz JM (2002). The utility of Ki-67 and BrdU as proliferative markers of adult neurogenesis. J Neurosci Methods.

[64] Tan S, Schubert D, Maher P (2001). Oxytosis: a novel form of programmed cell death. Curr Top Med Chem.

[65] Yu P, Zhang X, Liu N (2021). Pyroptosis: mechanisms and diseases. Signal Transduct Target Ther.

[66] Maier T, Güell M, Serrano L (2009). Correlation of mRNA and protein in complex biological samples. FEBS Lett.

[67] Brenchat A, Nadal X, Romero L (2010). Pharmacological activation of 5-HT_7_ receptors reduces nerve injury-induced mechanical and thermal hypersensitivity. Pain.

[68] Feng C, Luo T, Zhang S (2016). Lycopene protects human SHSY5Y neuroblastoma cells against hydrogen –peroxide induced death via inhibition of oxidative stress and –mitochondria associated apoptotic pathways. Mol Med Rep.

[69] Chwastek J, Jantas D, Lasoń W (2017). The ATM kinase inhibitor KU-55933 provides neuroprotection against hydrogen peroxide-induced cell damage via a γH2AX/p-p53/caspase-3-independent mechanism: Inhibition of calpain and cathepsin D. Int J Biochem Cell Biol.

[70] Krobert KA, Andressen KW, Levy FO (2006). Heterologous desensitization is evoked by both agonist and antagonist stimulation of the human 5-HT_7_ serotonin receptor. Eur J Pharmacol.

[71] Parga J, Rodriguez-Pallares J, Munoz A (2007). Serotonin decreases generation of dopaminergic neurons from mesencephalic precursors via serotonin type 7 and type 4 receptors. Dev Neurobiol.

[72] Zhang QJ, Du CX, Tan HH (2015). Activation and blockade of serotonin7 receptors in the prelimbic cortex regulate depressive-like behaviors in a 6-hydroxydopamine-induced Parkinson’s disease rat model. Neuroscience.

[73] Han LN, Zhang L, Sun YN (2016). Serotonin7 receptors in the lateral habenular nucleus regulate depressive-like behaviors in the hemiparkinsonian rats. Brain Res.

[74] Kvachnina E, Liu G, Dityatev A (2005). 5-HT_7_ receptor is coupled to Gα subunits of heterotrimeric G12-protein to regulate gene transcription and neuronal morphology. J Neurosci.

