# Supplementary material for: Neuroprotective and Neurite Outgrowth Stimulating Effects of New Low-Basicity 5-HT7 Receptor Agonists: In Vitro Study in Human Neuroblastoma SH-SY5Y Cells
Source: Neurochem Res. 2024 Jun 4;49(8):2179–96. doi: 10.1007/s11064-024-04159-z (PMC11233329; doi:10.1007/s11064-024-04159-z)
Supplement: Supplementary file 1 — Supplementary file1 (DOCX 1128 KB) [file 11064_2024_4159_MOESM1_ESM.docx]

**Supplementary data**

**Table S1.** Model of cell damage evoked by H_2_O_2_ in UN-SH-SY5Y cells cultured in DMEM and NB experimental medium and in RA-SH-SY5Y cells cultured in NB experimental medium.

|  | UN-SH-SY5Y in DMEM | | UN-SH-SY5Y in NB | | | RA-SH-SY5Y in NB | | |  |
| --- | --- | --- | --- | --- | --- | --- | --- | --- | --- |
|  | WST-1 | LDH | | WST-1 | LDH | | WST-1 | LDH | |
| control | 100.00 | 100.00 | | 100.00 | 100.00 | | 100.00 | 100.00 | |
| H_2_O_2_ | 50.78±0.61 ** | 378.17±0.80 *** | | 68.92±0.22 *** | 398.60±0.99 *** | | 65.93±0.36 *** | 406.67±0.67 *** | |
| H_2_O_2_ + NAC | 99.70±17.02 ## | 277.65±22.95 **, ### | | 109.75±4.37 ### | 290.69±17.10 ***, ### | | 97.61±3.57 ### | 305.17±69.35 ** | |
| N | 4-5 | 3 | | 8-9 | 3 | | 6 | 4 | |

The cells were treated with H_2_O_2_ (375 µM for UN-/DMEM, 150 μM for UN-/NB and 200 µM for RA-/NB) alone or in combination with NAC (1 mM) for 24 h. Cell damage were measured with cell viability (WST-1 and LDH release assay). Results were normalized to control group and are presented as the mean ± S.E.M. Data from 3-9 independent experiments were analyzed by one-way ANOVA and Duncan’s posthoc test. ^**^p<0.01 and ^***^p<0.001 *vs.* control group; ^##^p<0.01 and ^###^p<0.001 *vs.* H_2_O_2_ group.

|  | UN-SH-SY5Y | RA-SH-SY5Y |
| --- | --- | --- |
| control | 100.00 | 100.00 |
| 6-OHDA | 46.84 ± 0.19 *** | 65.55 ± 5.10 *** |
| 6-OHDA + NAC | 87.01 ± 5.44 **, ### | 94.62 ± 6.59 ### |
| N | 5-6 | 7 |

**Table S2.** Model of UN- and RA-SH-SY5Y cells damage evoked 6-OHDA in NB experimental medium.

Cells were treated with 6-OHDA (75 or 150 µM for UN- and RA-SH-SY5Y cells, respectively) or 6-OHDA+NAC (1 mM) for 24 h. Cell viability was measured with WST-1 test. Results were normalized to control and presented as mean ± S.E.M of 5-7 independent experiments. Data were analyzed by one-way ANOVA and Duncan’s post hoc test. ^**^p<0.01, ^***^p<0,001 *vs.* control group; ^###^p<0.001 *vs.* 6-OHDA group.

**Table S3.** The effects of 5-HT_7_ agonists against cell damage evoked by 6-OHDA in RA-SH-SY5Y cells cultured in NB experimental medium.

|  | WST-1 | LDH |
| --- | --- | --- |
| control | 100.00 | 100.00 |
| 6-OHDA | 66.17 ± 0.24 *** | 733.73 ± 97.35 *** |
| 5-CT 0.01 +  0.1 +  1 + | 66.42 ± 2.00 ***  67.12 ± 2.06 ***  68.18 ± 4.84 *** | 691.21 ± 67.21 ***  695.34 ± 53.28 ***  708.90 ± 70.54 *** |
| AH-494 0.01 +  0.1 +  1 + | 72.25 ± 4.55 ***  72.13 ± 2.04 ***  69.46 ± 2.67 *** | 737.12 ± 81.69 ***  741.97 ± 81.75 ***  834.93 ± 101.71 *** |
| AGH-238 0.01 +  0.1 +  1 + | 71.94 ± 2.35 ***  71.95 ± 4.68 ***  65.41 ± 4.07 *** | 706.76 ± 108.36 **  783.88±153.74 **  941.25±245.19 ** |
| AGH-194 0.01 +  0.1 +  1 + | 68.81 ± 3.04 ***  68.98 ± 3.03 ***  64.64 ± 2.78 *** | 636.79 ± 58.70 **  661.21 ± 102.46 **  768.52 ± 137.08 *** |
| N | 4-7 | 2-3 |

Cells were incubated for 30 min with 5-CT, AH-494, AGH-238 and AGH-194 at concentrations 0.01-1 µM followed by 24 h treatment with 6-OHDA (150 µM). Cell viability and cell cytotoxicity were measured WST-1 and LDH release assays, respectively. Results were normalized to control and presented as a mean ± S.E.M of 2-7 independent experiments. Data were analyzed by one-way ANOVA and Duncan’s post hoc test. ^**^p<0.01 and ^***^p<0.001 *vs.* control group.

**Table S4.** The effects of 5-HT_7_ agonists against cell damage induced by MPP+ in UN- and RA-SH-SY5Y cells cultured in NB experimental medium.

|  | UN-SH-SY5Y | RA-SH-SY5Y |
| --- | --- | --- |
| control | 100.00 | 99.89 ± 1.12 |
| MPP+ | 52.16 ± 0.08 *** | 44.60 ± 0.18 *** |
| 5-CT 0.01 +  0.1 +  1 + | 54.69 ± 2.48 ***  51.89 ± 2.56 ***  50.83 ± 3.81 *** | 38.90 ± 3.22 ***  36.83 ± 3.48 ***  43.14 ± 4.48 *** |
| AH-494 0.01 +  0.1 +  1 + | 63.59 ± 6.62 ***  57.25 ± 4.15 ***  57.46 ± 4.14 *** | 41.41 ± 6.28 ***  49.14 ± 5.61 ***  42.08 ± 9.10 *** |
| AGH-238 0.01 +  0.1 +  1 + | 54.91 ± 3.27 ***  52.10 ± 3.84 ***  51.69 ± 2.24 *** | 37.74 ± 6.22 ***  38.27 ± 3.48 ***  40.14 ± 4.62 *** |
| AGH-194 0.01 +  0.1 +  1 + | 62.35 ± 5.62 ***  61.12 ± 6.50 ***  57.22 ± 3.33 *** | 43.48 ± 7.12 ***  42.38 ± 9.79 ***  49.09 ± 10.43 *** |
| N | 6 | 4 |

Cells were incubated for 30 min with 5-CT, AH-494, AGH-238 and AGH-194 at concentrations 0.01-1 µM followed by 48 h treatment with MPP+ (1 and 3 mM for UN- and RA-SH-SY5Y cells, respectively). Cell viability and cytotoxicity were measured by WST-1 and LDH release assays, respectively. Results were normalized to control and presented as a mean ± S.E.M of 4-6 independent experiments. Data were analyzed by one-way ANOVA and Duncan’s post hoc test. ^***^p<0.001 *vs.* control group.

**Table S5.** The effects of 5-HT_7_ agonists against cell damage induced by doxorubicin (Dox) in UN- and RA-SH-SY5Y cells cultured in NB experimental medium.

|  | UN-SH-SY5Y | RA-SH-SY5Y |
| --- | --- | --- |
| control | 100.00 | 100.00 |
| Dox | 61.61 ± 0.90 *** | 51.50 ± 0.23 *** |
| 5-CT 0.01 +  0.1 +  1 + | 67.08 ± 4.64 ***  64.93 ± 1.54 ***  61.15 ± 1.79 *** | 57.33 ± 2.35 ***  56.43 ± 5.03 ***  56.76 ± 4.38 *** |
| AH-494 0.01 +  0.1 +  1 + | 67.20 ± 3.78 ***  60.82 ± 2.46 ***  60.27 ± 1.29 *** | 60.92 ± 6.80 ***  59.48 ± 4.58 ***  52.10 ± 3.90 *** |
| AGH-238 0.01 +  0.1 +  1 + | 61.93 ± 1.71 ***  59.44 ± 3.69 ***  60.80 ± 3.92 *** | 54.23 ± 3.74 ***  53.03 ± 6.57 ***  56.69 ± 6.34 *** |
| AGH-194 0.01 +  0.1 +  1 + | 59.68 ± 1.44 ***  60.04 ± 2.01 ***  57.60 ± 2.17 *** | 55.67 ± 3.68 ***  55.85 ± 2.00 ***  50.72 ± 0.96 *** |
| N | 5 | 4 |

Cells were pretreated for 30 min with 5-CT, AH-494, AGH-238 and AGH-194 at in concentrations 0.01- 1 µM followed by 24 h of treatment with Dox (1 and 2 µM for UN- and RA-SHSY5Y, respectively). Cell viability was measured by WST-1 test. Results were normalized to control and presented as a mean ± S.E.M of 4-5 independent experiments. Data were analyzed by one-way ANOVA and Duncan’s post hoc test. ^***^p<0.001 *vs.* control group.

**Figure S1.** DIC (Differential Interference Contrast) microphotographs of UN-SH-SY5Y and RA-SH-SY5Y cells cultured in NB experimental medium and treated with an antioxidant N-acetyl-cysteine (NAC) and H_2_O_2_. Cells were co-treated with NAC (1 mM) and H_2_O_2_ (150 µM and 200 mM for UN- and RA-SH-SY5Y cells, respectively) for 24 h.

UN-SH-SY5Y

RA-SH-SY5Y


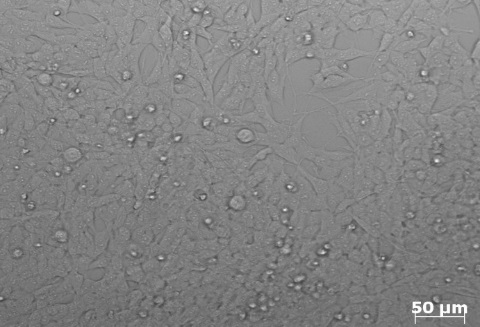


control


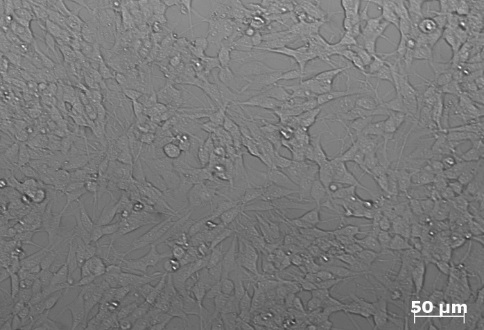


control


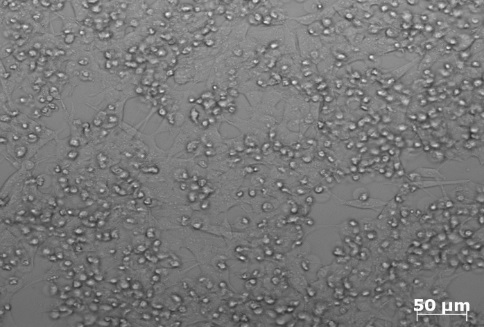


H_2_O_2_


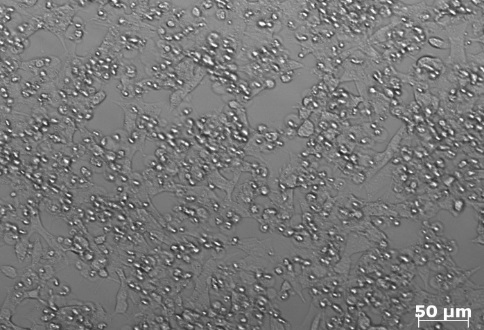


H_2_O_2_


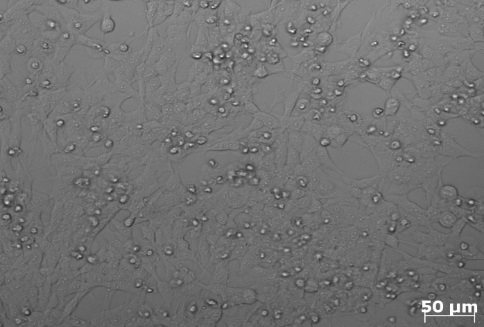


H_2_O_2_+NAC


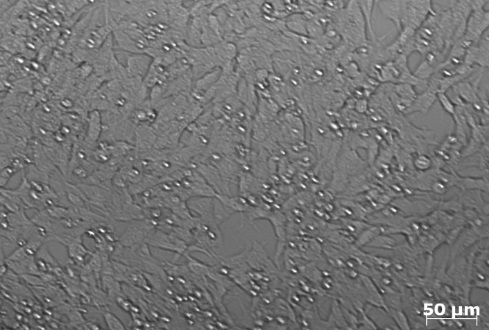


H_2_O_2_+NAC
